# Robust functional nanohybrid surface enables long-term oily wastewater remediation

**DOI:** 10.1186/s40580-025-00527-9

**Published:** 2025-12-23

**Authors:** Zheng Chen, Xuanyu Zhou, Hongyuan Zhang, Yuxiang Xue, Nurul Ain Mazlan, Siyu Chen, Xiuming Wei, Xianfeng Chen, Yi Huang

**Affiliations:** 1 Institute for Materials & Processes, School of Engineering, The University of Edinburgh, Robert Stevenson Road, Edinburgh, EH9 3FB UK; 2 Institute for Bioengineering, School of Engineering, The University of Edinburgh, Faraday Building, Edinburgh, EH9 3DW UK

**Keywords:** Nanohybrid, Superhydrophilic surface, Long-term stability, Robust separation performance, Oily wastewater remediation

## Abstract

Robust and scalable materials for oil-water separation are often limited by corrosion, fouling, and mechanical damage. Here, we demonstrate a simple, rapid, and low-temperature surface conversion that transforms commercial 316L stainless-steel meshes into superhydrophilic, underwater-oleophobic filters. A two-step reaction followed by Fe^3+^-tannic acid-phosphate complexation yields a thin nanogranular FeOOH/TA/PO_4_ hybrid film that is chemically anchored to the steel surface. The coating imparts rapid wetting behaviour, with a static water contact angle of about 45° and dynamic superhydrophilicity that achieves complete wetting within 0.15 s, enabling high-performance gravity-driven separations. Single-pass treatment of diverse oil-water mixtures delivered oil rejections of 95–99% and water permeate fluxes in the range of 23,000–80,000 L m^–2^ h^–1^, depending on mesh size. The meshes retained a robust performance over 50 separation cycles, 30 days of brine immersion, and exposures across pH 1–14 with negligible degradation. Abrasion resistance was further validated, and fouled meshes (e.g., with graphene oxide or edible oil) rapidly regained high flux and rejection after simple water rinsing, underscoring intrinsic self-cleaning behaviour. The process uses inexpensive reagents and is readily applicable to large-area mesh surface functionalization, providing a robust pathway to high-throughput, energy-free gravity separations for oily wastewater remediation.

**Graphical abstract:**

A simple, scalable two-step conversion of 316L stainless-steel mesh into a superhydrophilic filter coated with a robust protective nanohybrid film, which enables ultrafast oil-water separation, strong abrasion and anti-fouling resistance, corrosion tolerance, and long-term durability. Background illustration was created using *Midjourney*, a text-to-image AI tool.
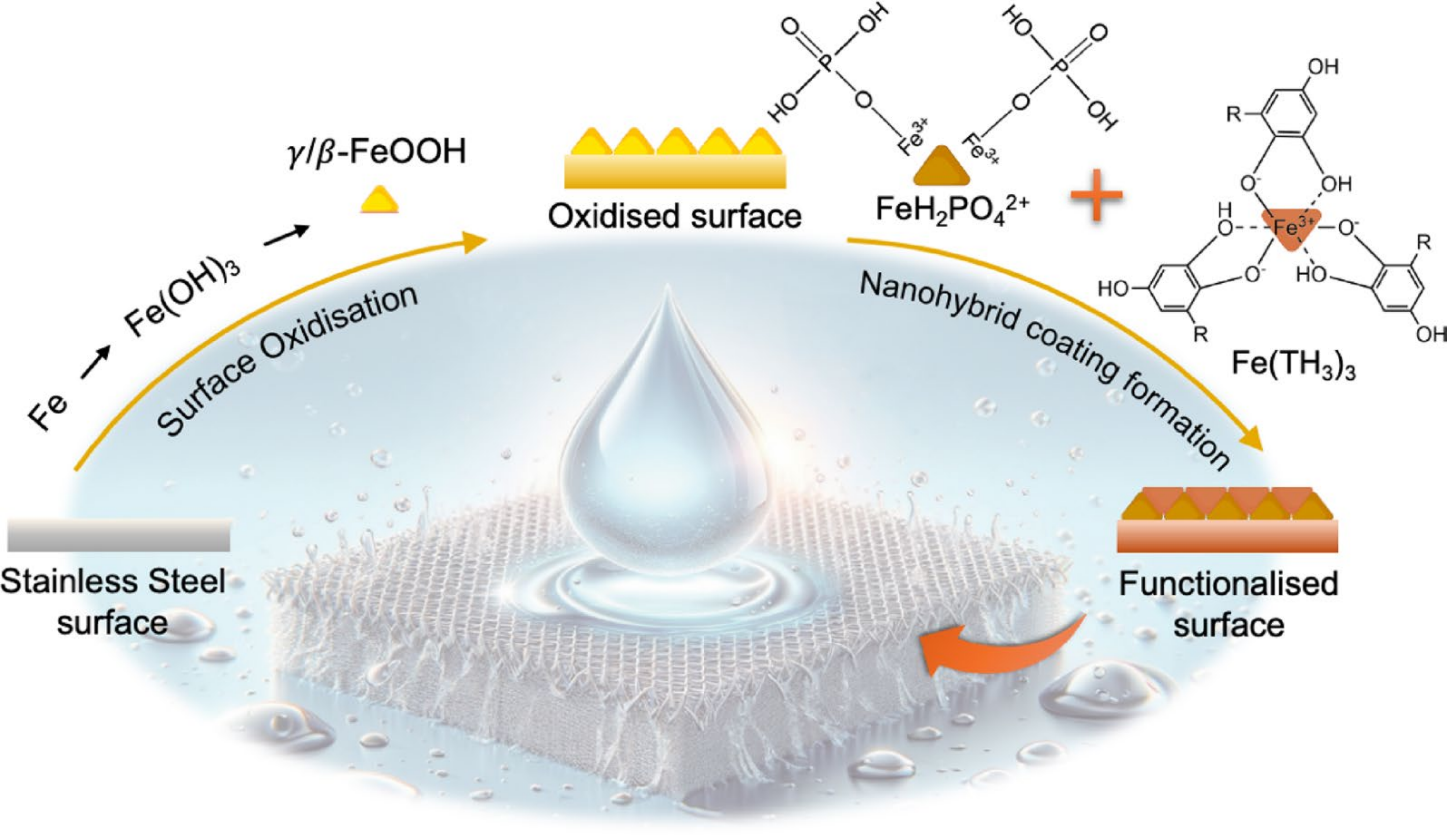

**Supplementary Information:**

The online version contains supplementary material available at 10.1186/s40580-025-00527-9.

## Highlights

Designing a swift, scalable treatment for facile steel surface modification.

Functional nanohybrid surface enables strong superhydrophilicity and chemical stability.

Effective separation of diverse oily waste mixtures with exceptional performance.

Long-term durability and remarkable resistance to corrosion, abrasion, and surface fouling.

**Supplementary Information:**

The online version contains supplementary material available at 10.1186/s40580-025-00527-9.

## Introduction

Oil-water separation technology plays a crucial role in environmental protection and industrial production. With the acceleration of industrialisation, the production of oil-water mixtures has significantly increased [1]. For instance, the iron and steel industries generate significant quantities of alkaline oily wastewater during various manufacturing processes, including annealing, pickling, cold rolling, and grinding [2]. This is not only derived from industrial processes, such as petroleum refining and chemical manufacturing, but also includes wastewater treatment in daily life, for example, in the catering industry and residential sewage [3–5]. Improper handling of oil-water mixtures can cause severe environmental pollution, adversely affecting the quality of water resources and the balance of ecosystems [6]. Hence, there is a growing demand to develop robust treatment strategies to achieve efficient and environmentally friendly management of spilled oil or oil-water mixtures [7, 8].

Early oil-water separation technologies primarily relied on physical separation principles, such as utilising density differences for gravity separation [9]. However, these methods were less efficient when dealing with complex oil-water mixtures [10]. Subsequently, researchers began exploring more efficient techniques, including centrifugal separation [11], adsorption [12], and flocculation [13, 14]. In recent years, with the advancement of material science, a series of new oil-water separation technologies based on nanotechnology and advanced materials have emerged, such as superhydrophobic and superhydrophilic surface treatment technologies [15]. However, simple and more sustainable technologies are still in high demand for the preparation of low-resource materials that are robust in both chemical stability and long-term separation performance.

Superhydrophilic surface treatments typically involve introducing polar groups onto the material surface or enhancing the surface hydrophilicity through the construction of nano-level microstructures [16]. These modifications can be achieved in various ways, including chemical vapor deposition (CVD) [17], sol-gel processes [18], and electrochemical deposition [19]. These methods formed nano-scale rough structures on the material surface, substantially increasing the contact area between the surface and water molecules and thereby enhancing hydrophilicity. Currently, superhydrophilic surface treatment technology has been applied in multiple fields. In industrial wastewater treatment, this technology effectively separates oil and harmful chemicals, protecting water resources [20]. In the emergency response to offshore oil spills, superhydrophilic materials demonstrated great potential due to their efficient oil-water separation capabilities [21]. Additionally, this technology has also found applications in industries such as food processing and carbon capture [22, 23].

Although superhydrophilic surface treatment technology excels in oil-water separation, it still faces several challenges. One of these is the long-term stability and durability of materials, especially under harsh environmental conditions [13, 24, 25]. Additionally, large-scale production and cost-effectiveness are also challenges faced by the current technology. Future research may focus on improving the stability and durability of materials and finding more economical and effective preparation methods. Recently, the study conducted by Shin et al. explored a superhydrophilic steel mesh created *via* a one-step annealing process, demonstrating its ability to maintain reusability even after 30 cycles [26]. Aditya et al. presented findings on a superhydrophilic mesh featuring a rough and uneven surface texture, achieved through a straightforward chemical etching method. This mesh exhibited the ability to withstand abrasion for a distance of at least 13.2 m under a 0.2 N weight load and resist corrosion when exposed to solutions with a pH of 2 or 13 [27]. Khosravi et al.developed a superhydrophilic mesh that was dip-coated with carbon soot and a polypyrrole/stearic acid film. This mesh demonstrated the capability to withstand corrosion within a pH range of 1.7 to 11 and exhibited exceptional durability for up to 30 cycles [25].

In this study, a superhydrophilic filter mesh (SHM) designed for oil-water separation was created utilising a range of commercial stainless-steel meshes. The process involved a controlled mesh surface oxidation step using a mixed solution of ammonium persulfate ((NH_4_)_2_S_2_O_8_) and sodium hydroxide (NaOH), followed by a conversion step achieved by immersing them in a conversion solution containing tannic acid and phosphoric acid. The amphiphilic stainless-steel mesh, known for its high compressive strength, resistance to chemical erosion, and resistance to water staining, has found applications in water treatment [28]. Tannic acid, being a natural polyphenol, can form complexes with iron ions and induce hydrophilic modification because of its abundant hydroxyl (OH) groups [29]. This modification helped to maintain the special surface wettability over an extended period. This approach offered the advantages of being simple, highly efficient, and conducted under mild conditions. Furthermore, long-term durability was assessed through a 30-day immersion in brine seawater. Severe corrosion and abrasion conditions were applied to assess the corrosion and abrasion resistance of the modified mesh. These measurements validated the successful preparation of SHMs, showcasing their superhydrophilic properties, along with stable oil-water separation efficiency and resistance to fouling. The energy-efficient, time-saving, and environmentally friendly fabrication process developed in this study holds significant potential for upscaling the production of superhydrophilic mesh, making it suitable for a wide range of applications in liquid mixture separation.

## Materials and methods

### Materials

Stainless-steel meshes (SSM, 316L grade; 300-mesh, aperture size: 50 μm; 400-mesh, aperture size: 35 μm; 500-mesh, apterture size: 27 μm) with a plain weave were purchased from Inoxia (Cranleigh, UK) Ltd. Cyclohexane (LR), dodecane (LR), toluene (LR), n-hexane (AR), ammonium persulfate (AR), sodium hydroxide (LR), tannic acid (AR), and phosphoric acid (AR) were purchased from Fisher Scientific UK Ltd. Pump oil was obtained from Thermo Fisher Scientific Co. (UK) Ltd. Mineral oil, machine oil, and paraffin oil were obtained from Sigma-Aldrich (Germany) Ltd. All the reagents were employed without any additional processing. Deionised water was acquired from laboratory water purification equipment with a conductivity of less than 1 µS cm^–1^.

### Superhydrophilic surface modification method

**Fig. 1:**
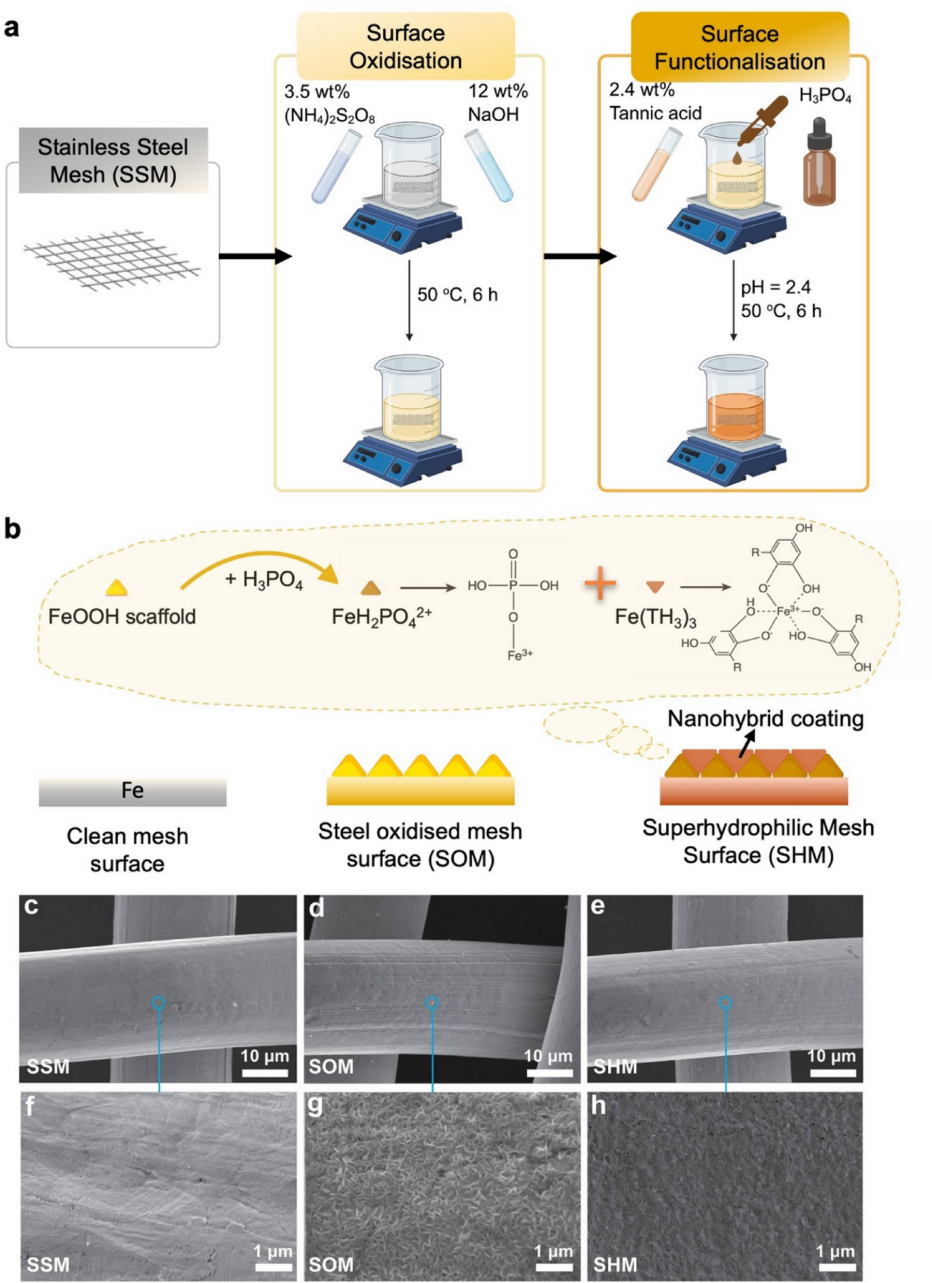
**Fabrication process of the superhydrophilic stainless steel mesh.** **a**, Workflow from a clean SSM to a SOM by immersion in 3.5 wt% (NH_4_)_2_S_2_O_8_ + 1.2 wt% NaOH at 50 ℃ for 6 h, followed by conversion to a SHM using tannic acid (2.4 wt%) and H_3_PO_4_ at pH 2.4, 50 ℃ for 6 h. **b**, Schematic illustration of the complexation process of FeOOH, TA, and H_3_PO_4_ during the conversion stage. The legends of meshes in different colours represented the colour changes of the mesh at different stages of the reaction. **c**–**e**, SEM overviews (scale bars, 10 μm) of (**c**) SSM, (**d**) SOM and (**e**) SHM; **f**–**h**, corresponding higher-magnification views of the regions indicated (scale bars, 1 μm). Oxidation roughened and corroded the wire surface (SOM), whereas the conversion step yielded a conformal phosphate-tannate/Fe hybrid coating (SHM) with a more uniform, nanogranular texture.

The stainless-steel mesh measuring 3 cm × 6 cm was first subjected to ultrasonic cleaning using acetone and ethanol to eliminate surface impurities. Subsequently, it was immersed in a 0.1 M hydrochloric acid solution and then rinsed with deionised water to eliminate surface oxide. Then, it was submerged into the oxidation solution and conversion solution that had been prepared in advance to complete the modification process at 50 ^o^C, as depicted in Fig. 1a. The treatment solution was formulated following a previous study with minor adjustments [30]. Typically, a mixture of aqueous solutions containing 3.5 wt% (NH_4_)_2_S_2_O_8_ and 12.0 wt% NaOH was employed as the oxidation solution. The conversion aqueous solution consisted of 2.4 wt% tannic acid, with its pH adjusted to 2.4 using phosphoric acid. During the oxidation phase, the stainless-steel mesh (SSM), which had been previously cleaned, was transformed into a steel oxidised mesh (SOM), exhibiting a yellowish coloration resulting from the formation of FeOOH. In the conversion process, the ≡ Fe–OH groups within the FeOOH reacted with phosphoric acid, while the liberated Fe(III) ions were complexed by the phenolic hydroxyl groups present in tannic acid. This led to the creation of a violet-coloured superhydrophilic mesh (SHM). To ensure the trustworthiness and consistency of the experimental outcomes, three distinct series of samples were examined for various performance metrics. These metrics encompassed oil-water separation efficiency, long-term durability, corrosion resistance, abrasion resistance, and anti-fouling capabilities.

### Characterisation of mesh structure

The surface morphology of stainless-steel mesh was examined using a scanning electron microscope (SEM, Zeiss 400 Compact). The water contact angle (WCA) measurements were measured by a goniometer system (Ossila, Sheffield, UK) to evaluate the surface wettability of the mesh. Attenuated total reflection (ATR)-Fourier transform infrared (FTIR) analysis was performed using a Nicolet Summit X spectrometer to evaluate the surface functional groups and other chemical bonds. Further information regarding the chemical compositions of the modified stainless-steel mesh was analysed using both an EDS instrument (Oxford Instruments) and XPS (Kratos Axis Ultra DLD spectrometer). The crystal form of iron oxide of modified stainless-steel mesh was also characterised by an X-ray diffractometer with Cu Kα emission in a 2θ range of 10.0° to 50.0° (XRD, Bruker D6 Advance). The remaining oil content in the permeate solutions was assessed using a TOC (Shimadzu) analyser.

### Oil-water separation

The effectiveness of the modified superhydrophilic mesh in separating oil-water mixtures was assessed through a set of gravity-powered tests. We created the oil-water mixtures by simply mixing them, and added methylene blue to the water and oil red O to the oils to facilitate visualisation. We assembled a custom separation apparatus, placing the mesh between two identical 25-mm diameter glass tubes, and securely clamped them together (as illustrated in Fig. S1). During standard separation tests, the oil-water mixture was poured directly onto the mesh, and the filtrate was collected while continuously monitoring its flow rate in real-time. For reliable oil-water separation test results, the mesh was initially saturated with water by pouring roughly 50 mL of deionised water onto it. Subsequently, an oil-water mixture (1:1 v v^–1^) was poured into the upper funnel, and the filtrate water was collected in a beaker. Following that, the mesh was removed and delicately agitated in methanol using forceps, allowing it to soak for 10 min, and then air-dried for 5 min. The cleaned mesh was subsequently subjected to another round of separation tests.

The water flux ($J,$ kL m^–2^ h^–1^) of the modified mesh during the separation was computed using the following Eq. 1.

1 $$ J= \frac{V}{A\cdot t}$$

Here, $$ V$$ (L) represented the volume of the permeated water, $$ A$$ (m^2^) was the effective area of the mesh, and $$ t$$ (h) signified the permeation time. Eight different types of oil (including n-hexane, toluene, dodecane, cyclohexane, pump oil, machine oil, mineral oil, and sunflower oil) were employed to assess the oil rejection capability of the modified mesh. Oil rejection was determined using the following Eq. 2. 

2 $$ R\left(\%\right)=\left(1- \frac{{C}_{p}}{{C}_{o}}\right) \times 100\%$$

In this equation, $$ {C}_{p}$$ represented the remaining oil concentration in the collected filtrate, while $$ {C}_{0}$$ signified the initial oil concentration in the feed oil-water mixture.

## Results and discussion

### Morphology and structure characterisation

The superhydrophilic mesh filters were fabricated using a two-step oxidation-conversion method, as displayed in the schematic diagram in Fig. 1a. In the first step, iron oxide (FeOOH)-forming oxidation was carried out; in the second step, the oxidised surface was converted into the mixture solution of tannic acid (TA)/phosphoric acid (H_3_PO_4_). Together, these steps drove a staged composition-structure transition consistent with Fig. 1b: an O-rich FeOOH scaffold formed first, followed by deposition of a carbon-rich hybrid overlayer that partially passivated the steel surface. Establishing a reproducible protocol was essential; therefore, pre-cleaning was performed to remove adventitious films/particulates and to expose the intrinsic metal texture (Fig. S2), thereby minimising roughness variability prior to chemical modification. As a result, the as-received stainless-steel mesh (SSM) displayed a clean, smooth surface in SEM, as depicted in Fig. 1c, f, and contained elemental percentages of carbon (C) at 10.1% and oxygen (O) at 9.5%, as determined by EDS analysis (Table 1). Oxidation at room temperature (R.T.), however, proved ineffective: even after 2–36 h, SEM revealed only sparse etch tracks at short times, progressing to discontinuous corrosion islands, pore-edge pitting and flake-like detachment, that is uncontrolled corrosion rather than uniform coverage (Fig. S3). By contrast, mild heating at 50 °C resolved this bottleneck. Nascent nanofibrils (after only 1–2 h) coalesced into a dense, interlaced FeOOH network by ≈ 6 h (Figs. 1d, g and S4), providing a coherent scaffold for the subsequent conversion. Elemental mapping tracked this build-up: relative to cleaned SSM (3.32% of O), SOM prepared at 50 °C for 1/2/6 h exhibited progressively higher and more uniform O fractions (10.62/15.94/28.61%), whereas the R.T. series remained heterogeneous (Fig. S5). High-precision EDS point analyses further confirmed a shift in surface composition, with C and O increasing to 20.9% and 33.9% (Table 1), respectively, attributable to the oxidation of iron. In parallel, ATR-FTIR captured the growth of surface hydroxyls: the ≈ 1967 cm^–1^ band assigned to ≡ Fe–OH intensified with temperature/time and was most pronounced at 50 °C-6 h (Fig. S6), consistent with progressive hydroxylation of FeOOH. XRD corroborated phase development: lepidocrocite (γ-FeOOH, L) and akaganeite (β-FeOOH, A) reflections sharpened under 50 °C oxidation and remained broad/weak at R.T., evidencing increased crystallinity/fraction only with mild heating (Fig. S7).

**Fig. 2:**
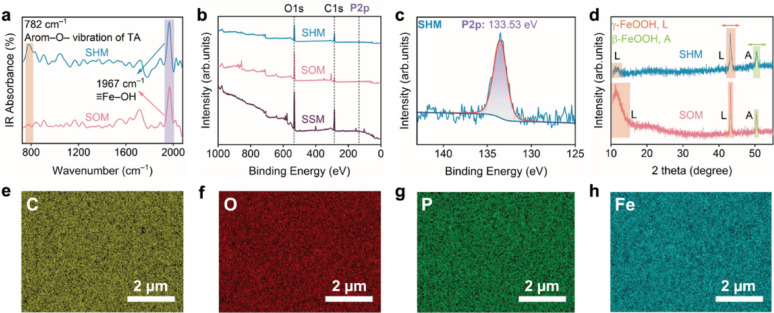
**Chemical signatures and phase evolution during oxidation–conversion of stainless-steel meshes.  a**, ATR-FTIR spectra comparing SOM and SHM, highlighting the ~ 782 cm^–1^ aromatic O^–^ vibration of tannic acid (TA) and the ~ 1967 cm^–1^ ≡Fe–OH band. **b**, XPS survey spectra of SSM, SOM, and SHM showing O1s, C1s, and P2p regions. **c**, High-resolution P2p spectrum of SHM with a component at 133.53 eV, consistent with phosphate incorporation. **d**, XRD patterns with reflections assigned to lepidocrocite (γ-FeOOH, L) and akaganeite (β-FeOOH, A); shaded bands marked their characteristic regions, with arrows indicating attenuated features in SHM. **e**–**h**, EDS elemental maps (SHM) for C, O, P and Fe, demonstrating uniform distribution of the hybrid coating across the surface. Scale bars, 2 µm.

The conversion step then required both tight acidity control and heat. Without precise control at pH 2.4, post-treated meshes retained a fibrillar-like FeOOH texture on SOM even after 50 °C-24 h or R.T.-24 h, lacking the continuous nanogranular film that defined SHM (Fig. S8). Consistently, the corresponding O-maps were modest (~ 12–18%) and spatially non-uniform (Fig. S9), evidencing inefficient Fe^3+^-tannic/phosphate complexation. Pure R.T. conversion was likewise ineffective: 6–12 h yielded worm-like, porous deposits with abundant sub-micrometre voids (Fig. S10). In contrast, at 50 °C the surface evolved along a clear pathway—from nucleation/coalescence (1–2 h) to a dense, conformal nanogranular film by ≈ 6 h, whereas prolonging to 12 h induced coarsening and local pore infilling (Figs. 1e, h and S11). Notably, the apparent O-map intensity reached a minimum at 50 °C-6 h (≈ 3.10%) and increased again for shorter/longer or R.T. conversions (Fig. S12). In line with this, elemental point analysis revealed a concomitant rise in surface carbon content (to 39.3%) and a decrease in oxygen content (to 23.3%). This phenomenon was readily rationalized by the TA-derived coating: tannic acid was a polyphenol with a high C/O ratio (aromatic rings and galloyl groups), so formation of a compact hybrid overlayer introduced more carbon-rich organic material while partially masking the underlying FeOOH and attenuating its detectable O signal [31, 32]. Chemical fingerprints aligned with this window: the TA-associated aromatic O^–^ vibration (~ 780–810 cm^–1^) was strongest at 50 °C-6 h and weak at R.T.-12 h (Fig. S13); diffraction showed that SHM dismissed the low-angle L band (≈ 11.15°) and displayed attenuated L (≈ 43°)/A (≈ 51°) peaks relative to SOM (Fig. S14), a suppression emphasised by the direct SOM↔SHM comparison (Fig. 2d) [33, 34]. Relative to SOM, SHM showed the disappearance of the low-angle L band (~ 10–15°) and reduced intensities of the L (≈ 43°) and A (≈ 51°) peaks. This suppression evidenced a lower exposed/crystalline FeOOH fraction after conversion, consistent with coverage by a more amorphous phosphate-tannate hybrid layer that passivated the surface. XPS further confirmed the conversion chemistry: the presence of a clear P2p component at 133.53 eV (Fig. 2b, c and Table S1) was consistent with phosphate incorporation from H_3_PO_4_ during pH control. Finally, side-by-side FTIR (SOM vs. SHM) showed a pronounced ≈ 782 cm^–1^ TA band alongside a concomitantly weaker ≡ Fe–OH envelope after conversion (Fig. 2a), indicating that while TA/phosphate grafting was successful, some FeOOH residues persisted on the wire framework [35]. Altogether, the sequence from Figs. S1–S14, together with Figs. 1 and 2; Table 1 and S1, delineated a narrow but robust process window—oxidation at 50 °C followed by conversion at pH 2.4 for ≈ 6 h—that yielded a chemically integrated phosphate-tannate/Fe network, suppressed exposed/crystalline FeOOH signatures and imparted the uniform nanogranular SHM texture that underpinned the wetting and separation behaviour discussed next.

**Table 1:** Surface elemental composition of stainless-steel meshes at each modification stage

Sample	Atomic Percentage (%)				Weight Percentage (%)			
	C	O	Fe	Ni	C	O	Fe	Ni
SSM	10.1	9.5	68.6	11.7	2.5	3.2	79.9	14.3
SOM	20.9	33.9	34.4	10.7	14.1	15.9	52.4	17.5
SHM	39.3	23.3	29.7	7.8	16.4	12.5	55.6	15.3

Based on the experimental results, the reaction processes for oxidation and conversion were extrapolated. In the initial step, the oxidation of Fe occurred alongside the complexation of Fe^3+^ and OH^–^ ions, as illustrated in Eq. 3. Upon drying, Fe(OH)_3_ was transformed into FeOOH, as indicated in Eq. 4.

3 $$Fe+3O{H}^{-}\to Fe(OH{)}_{3}+3{e}^{-}$$

4 $$ Fe(OH{)}_{3}\to FeOOH+{H}_{2}O$$

In the second step, the complexation reaction between Fe^3+^ and tannic acid (TH_4_) was depicted in Eq. 5. The complexation between FeOOH and phosphoric acid was shown in Eq. 6.

5 $$F{e}^{3+}+T{H}_{4}\to Fe(T{H}_{3}{)}_{3}+3{H}^{+}$$

6 $$FeOOH+{H}_{3}P{O}_{4}\to Fe({H}_{2}P{O}_{4}{)}_{3}+{H}_{2}O$$

As shown in Fig. 1b, FeOOH clusters released Fe cations, which can then create a film by forming complexes with tannic acid in the acidic conversion solution. Simultaneously, a dense phosphating film was generated through the bonding of H_3_PO_4_ with FeOOH.

### Surface wettability of functionalised SHMs

**Fig. 3:**
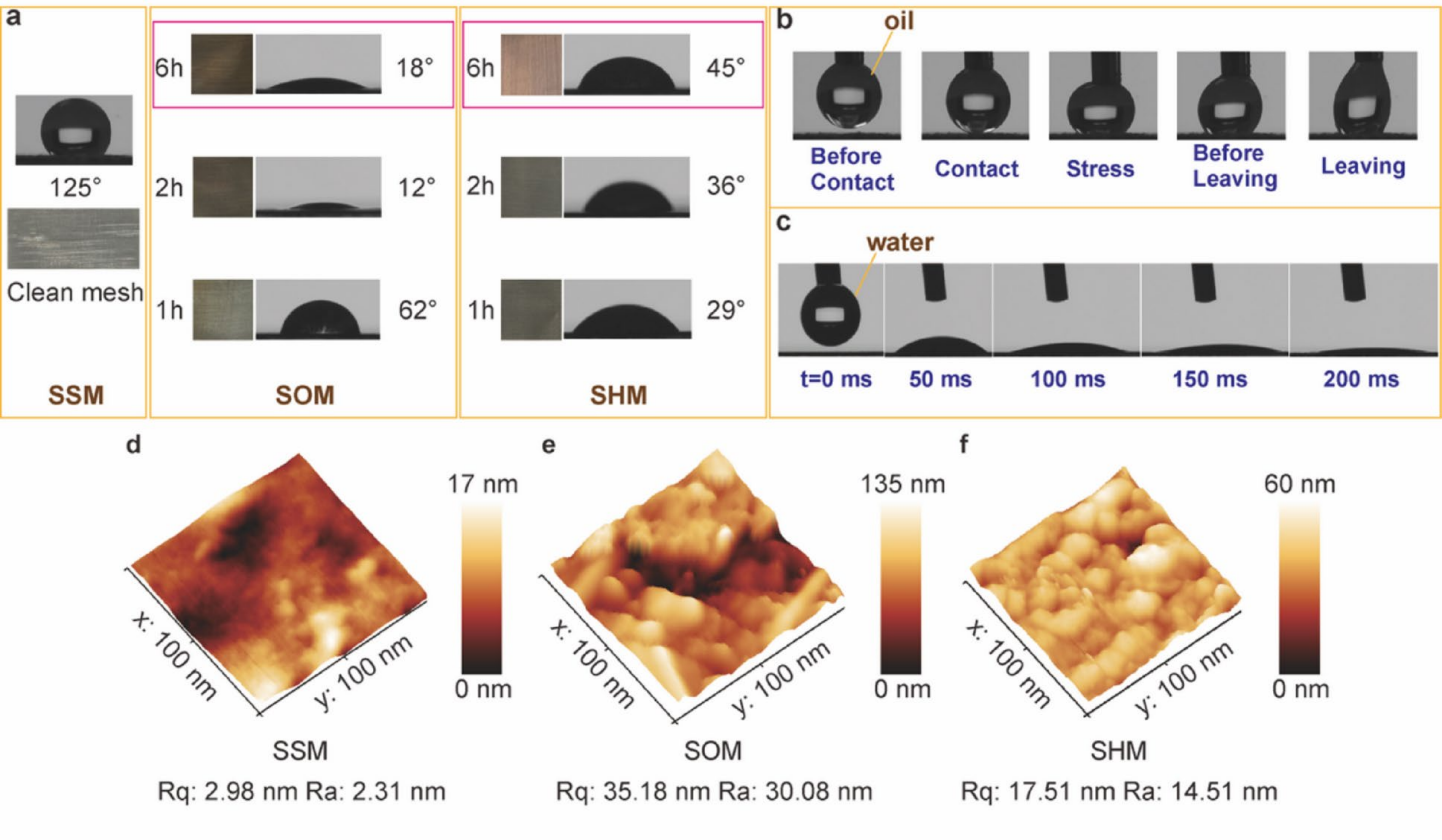
**Wetting transformation and nanoscale roughness during oxidation-conversion of stainless-steel meshes.** **a**, Photographs and static water contact angles for the clean mesh (SSM) and meshes after oxidation (SOM, 1–6 h) or subsequent conversion to the superhydrophilic state (SHM, 1–6 h). Oxidation markedly lowered the water angle (e.g., 62° → 18° → 12° for 1–6 h SOM), while the converted SHM exhibited uniformly low angles (36–64°). **b**, Contact-release sequence of a 2 µL toluene droplet on the SHM surface, showing low adhesion and clean detachment. **c**, Time-resolved spreading of a 2 µL water droplet on SHM (0–200 ms), evidencing rapid wetting consistent with superhydrophilicity. **d**–**f**, AFM topography and roughness evolution during mesh modification: [average surface roughness (Ra) and root-mean-square roughness (Rq)] (**d**) SSM, (**e**) SOM, and (**f**) SHM.

Subsequently, the surface wetting properties of the SSM, SOM, and SHM were analysed. Fig. 3a presented digital images and water contact angles (WCA) of the SSM, SOM, and SHM under different oxidation and conversion times, ranging from 1 to 6 h at 50 °C. It was noticeable that the SOM and SHM exhibited different colours corresponding to their respective modification times. Among them, although the SOM with 2 h oxidation showed the lowest WCA value of 12°, the difference was only 6 degrees compared to the 6 h sample. Therefore, considering the integrity of the hierarchical surface structure of SOM (Fig. S4), the 6 h sample was used for the following conversion experiment. The WCA values demonstrated a tendency to decrease as the oxidation time progressed, a phenomenon that could potentially be ascribed to alterations in surface roughness (Fig. 3d, e). Oxidation induced pronounced roughening, where the root mean square roughness (Rq) rose from 2.98 nm (clean mesh) to 35.18 nm with SOM (average surface roughness, Ra = 30.08 nm), yielding a hillock-like texture. This monotonic change was also consistent with the Wenzel relation, as shown in Eq. 7:

7 $$\text{cos}{\theta }^{*}=r\text{cos}{\theta}_{0}$$

where $${\theta}^{*}$$ is the apparent contact angle, $$r$$ is the surface roughness ratio, and $${\theta}_{0}$$ is the Young’s contact angle [36, 37]. The oxidation process increased the roughness factor of the mesh ($$ r$$ >1; Rq: 2.98 → 35.18 nm), which amplified the intrinsic hydrophilicity of FeOOH ($$ {\theta }_{0}$$ < 90°) and therefore lowered $$ {\theta }^{*}$$.

After an additional 6 h conversion, the resultant SHM exhibited a comparatively low WCA value of 45°, which was subsequently chosen as the optimal specimen for the subsequent experiment. Even though the 1 h sample exhibited a smaller water contact angle (29°), SEM results (Fig. S11) confirmed that a reaction time of 1 h was insufficient for the completion of the conversion reaction. The surface remained quite rough. To summarise, at the onset of the conversion process, the introduction of tannic and phosphoric acid enhanced the surface energy, resulting in a reduction in superhydrophilicity and a consequent increase in the WCA. With the prolongation of the conversion duration, the surface roughness diminished owing to the intensified protection from the coating layer, consequently leading to an upward trend in the WCA [38]. As shown in Fig. 3f, successful conversion to SHM partially smoothed the surface (Rq = 17.51 nm, Ra = 14.51 nm) while retaining nanoscale features consistent with a conformal hybrid coating. This increase in $${\theta }^{*}$$ relative to SOM was rationalised by two concurrent effects: (i) a reduction in $$ r$$ diminished Wenzel amplification, and (ii) grafting of the TA/phosphate overlayer moderated the surface polarity by masking a fraction of FeOOH –OH groups, effectively raising $$ {\theta }_{0}$$. Dynamic tests further supported this interpretation. As depicted in Fig. 3b, the toluene droplet readily detached from the SHM surface after being compressed against it under a specific pressure, signifying its minimal adhesion in an air environment. Fig. 3c illustrated the process of water droplet dispersion on the SHM surface. As demonstrated, the WCA can decrease to 0° within 150 ms of contact (Movie S1), indicative of the SHM’s superhydrophilic nature.

### Oil-water mixture separation performance

**Fig. 4:**
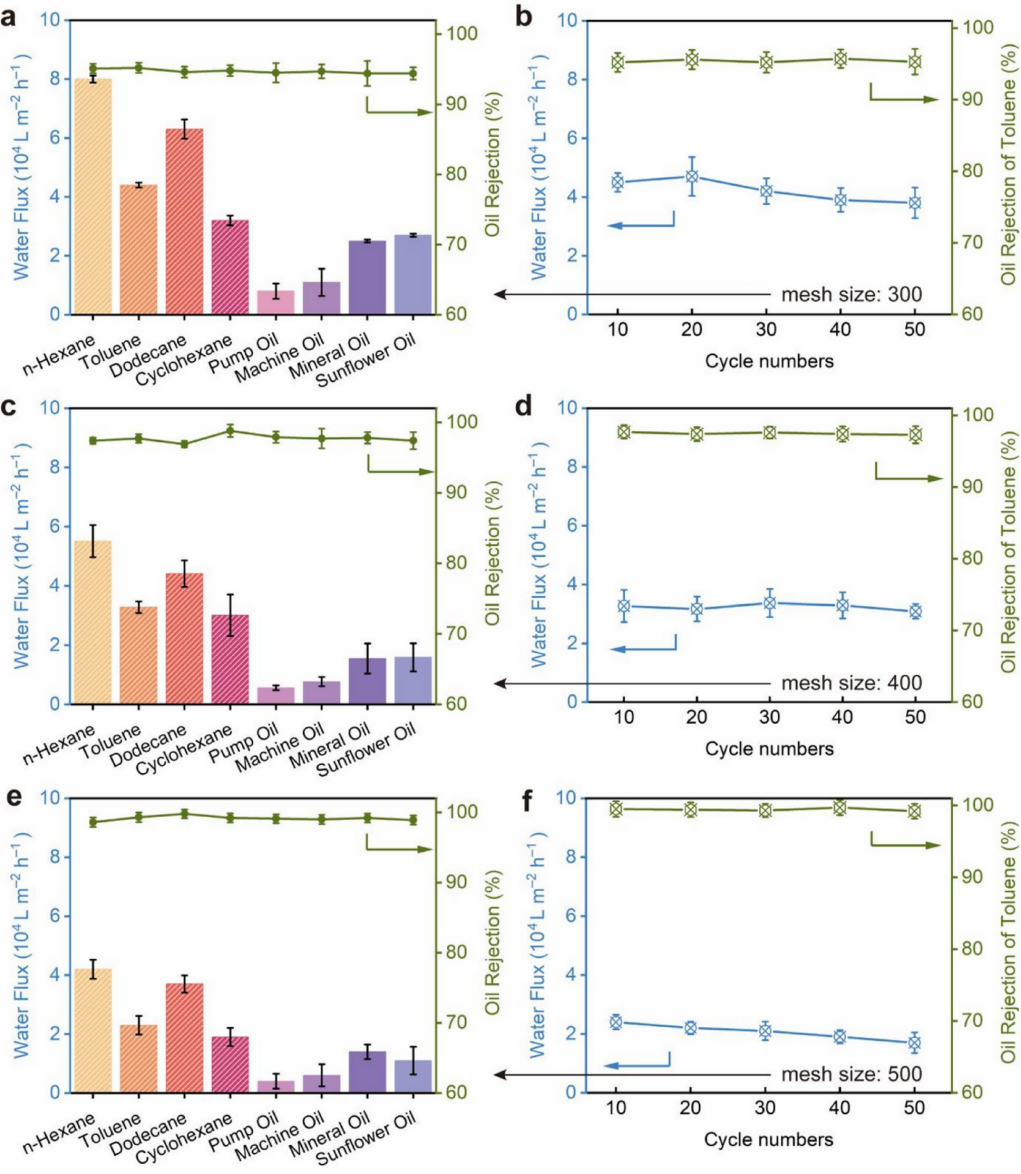
**Oil-water separation performance of superhydrophilic meshes (SHMs) with different mesh counts. a**, **c**, **e**, Single-pass separations of eight oil-water mixtures (n-hexane, toluene, dodecane, cyclohexane, pump oil, machine oil, mineral oil, sunflower oil) using (**a**) SHM-300, (**c**) SHM-400 and (**e**) SHM-500. Bars: water flux (left axis). Green markers/line: oil rejection (right axis). **b**, **d**, **f**, Cycling stability for the toluene/water mixture over 50 successive runs with (**b**) SHM-300, (**d**) SHM-400 and (**f**) SHM-500.

**Fig. 5:**
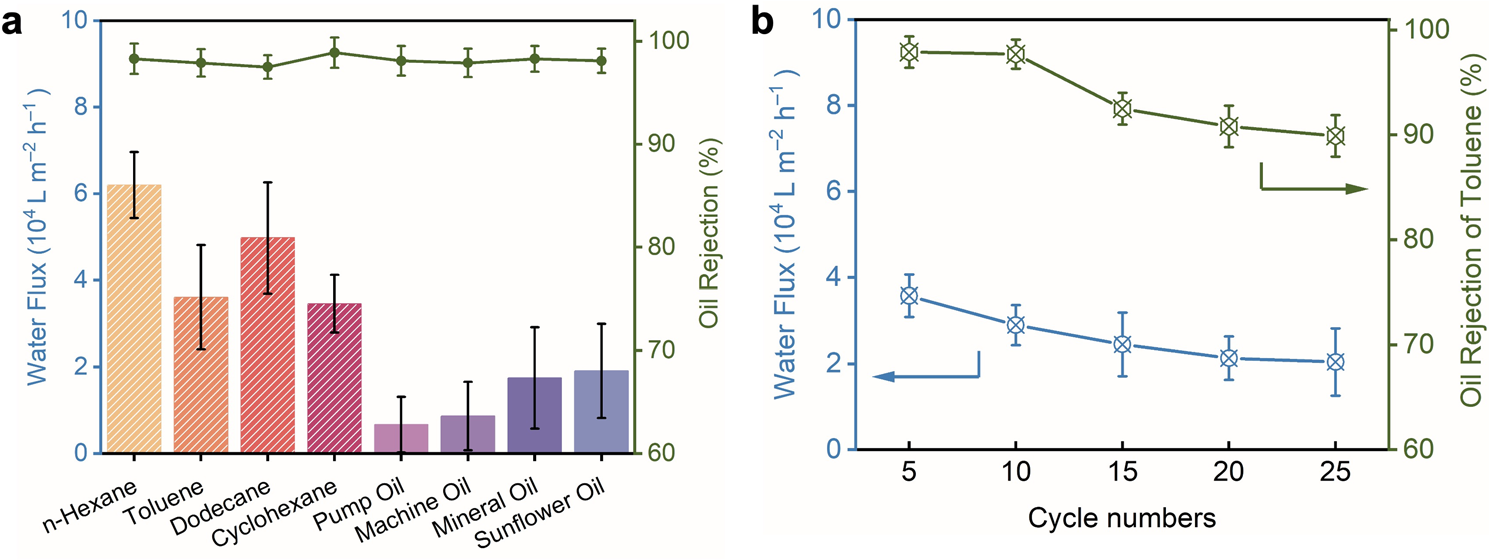
**Oil-water separation performance of the SOM-400 for eight different oil-water mixtures. a**, Single-pass separations of eight oil/water mixtures (n-hexane, toluene, dodecane, cyclohexane, pump oil, machine oil, mineral oil, sunflower oil). Bars: water flux (left axis, 10^4^ L m^–2^ h^–1^). Green markers/line: oil rejection (right axis, %). **b**, Cycling with a toluene/water mixture over repeated runs. Blue: water flux (left axis). Green: oil rejection (right axis).

In this study, the SHMs were utilised to execute oil-water separation processes with a variety of mixtures, specifically *n*-hexane, toluene, dodecane, cyclohexane, pump oil, machine oil, mineral oil, and sunflower oil, respectively (Movie S2). It was observable that water swiftly permeated the SHM, while the oil phase remained predominantly on the feed side. As shown in Fig. 4a, the water fluxes of the SHM-300 remained in the range of 8,000 – 80,000 L m^–2^ h^–1^ throughout the separation process of eight distinct oil-water mixtures. Furthermore, the rejection rates for oil in these various oil-water mixtures consistently exceeded 95.4%, signifying that the SHM effectively repelled the oil. Clearly, the application of oxidation and conversion techniques to the stainless-steel mesh granted the SHM remarkable efficiency in separating oil and water. This was in stark contrast to the SSM, which, without any modification, lacked the capacity to act as an oil-water separation mesh due to its inability to repel oil. Fig. 4b illustrates the separation efficacy of the toluene-water mixture across 50 iterations. Observations indicated that the water fluxes of the SHM oscillated around 40,000 L m^–2^ h^–1^, while the oil rejection rate consistently remained above 95.1% throughout 50 separation cycles. These findings demonstrated that the SHM possessed remarkable stability and was suitable for repeated use in the separation of oil and water. The effect of mesh count on separation performance was also evaluated. As shown in Fig. 4c, the permeate flux of SHM-400 experienced a reduction (from 5,600 to 55,000 L m^–2^ h^–1^), whereas the separation efficiency marginally rose from 95.4% to 97.4% upon utilising a mesh with a smaller pore size (400 counts, pore size: 35 μm). Cycling with a toluene-water mixture also demonstrated durability: SHM-400 maintained a flux of ≈ 31,000 L m^–2^ h^–1^ with > 97.3% rejection over 50 runs (Fig. 4d), indicating limited fouling and stable hydrophilicity. The oxidised control (SOM-400) also initially exhibited impressive efficiency in separating oil and water, achieving oil rejection rates exceeding 98.3% and maintaining a highest water flux of approximately 60,000 L m^–2^ h^–1^ (Fig. 5a). However, over multiple cycles, its flux showed an obvious decline to 89.9% rejection by only 25 cycles. Compared with SHM-400, the SOM samples, benefiting from its surface roughness exhibited superior separation performance; however, the absence of a protective coating compromised durability, leading to a pronounced decline in rejection after only five cycles (Fig. 5b). It is worthwhile mentioning that the efficiency of oil-water separation cannot be significantly enhanced further (Fig. 4e, f; Table 2), even by continuing increasing the counts of meshes to 500 (open apertures of 27 μm in size). Transitioning to a tighter mesh, SHM-500, produced only marginal additional gain in rejection (99.3%) while imposing a larger flux penalty (maximum flux: ≈ 40,000 L m^–2^ h^–1^); the improvement was not statistically significant relative to SHM-300/400. Additionally, employing a large-aperture mesh that was both cost-effective and mechanically robust holds significant promise in minimising capital expenditures. This was crucial for the development of large-scale applications involving this technology.

**Table 2:** Permeate flux and separation efficiency variations of mesh with different mesh sizes

Mesh count, aperture size	Water flux (L m^–2^ h^–1^)	Oil rejection of toluene (%)
300, 50 μm	44,000	95.2
400, 35 μm	33,000	97.7
500, 27 μm	23,000	99.3

### Sustained durability and chemical tolerance characteristics

**Fig. 6:**
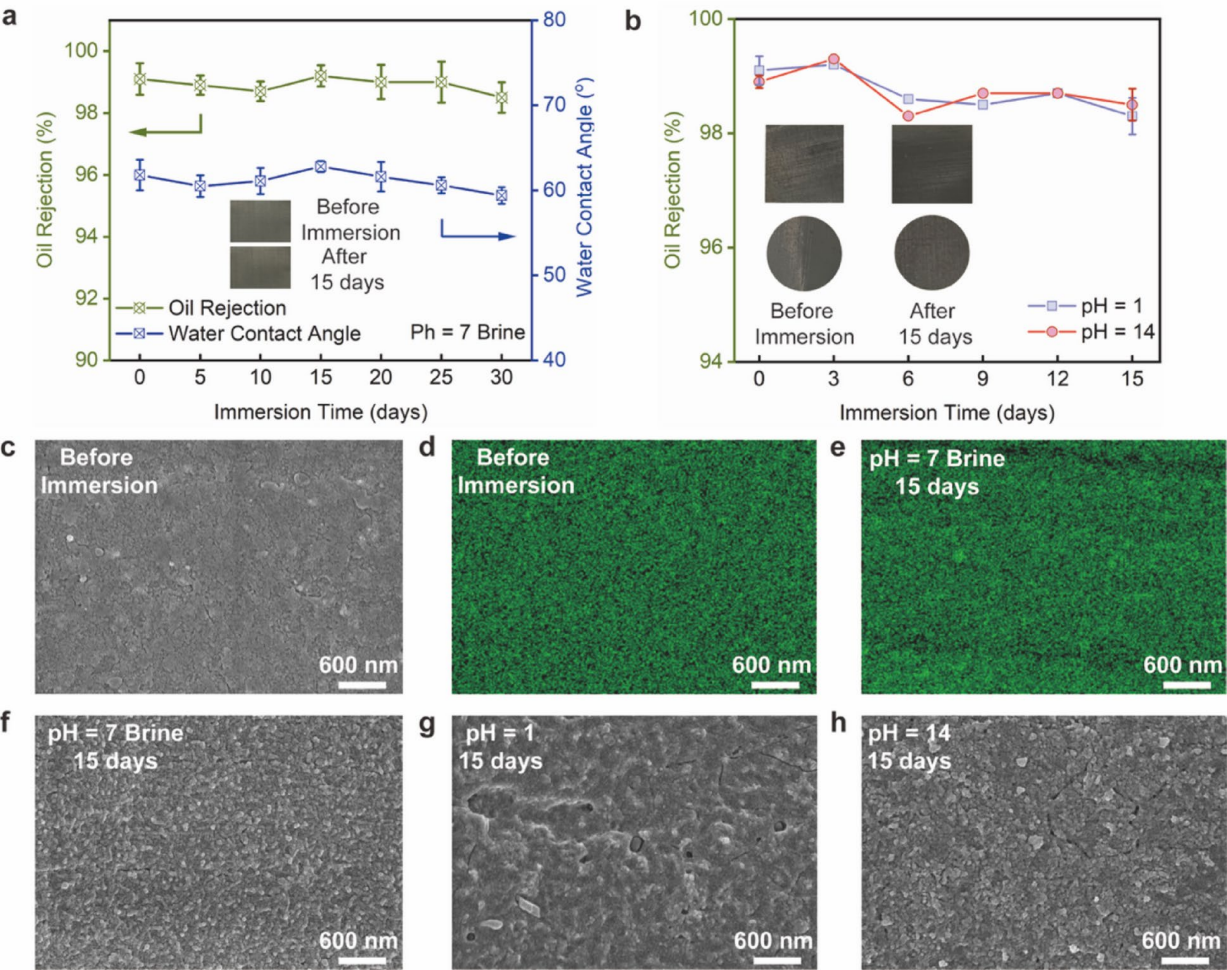
**Long-term chemical stability of the superhydrophilic mesh (SHM). a**, Evolution of oil rejection (left axis) and water contact angle (right axis) for SHM immersed in pH-neutral brine over 30 days; both metrics remained essentially constant. Photographs (insets) showed no visible degradation in 15 days. **b**, Oil rejection during immersion in strong acid (pH 1) and strong base (pH 14) for 15 days; photographs (insets) showed no visible degradation before/after soaking. **c**, High-magnification SEM image of SHM before immersion. **d**, **e**, Phosphorus maps of SHM: (**d**) before immersion and (**e**) after 15 days in pH 7 brine. **f**–**h**, High-magnification SEM images of SHM after 15 days in (**f**) pH 7 brine, (**g**) pH 1 acid, and (**h**) pH 14 base, revealing minimal morphological change. Scale bars, 600 nm.

To probe performance under chemically aggressive conditions, three immersion media were employed: pH-neutral artificial brine (composition in Table S2), a strong acid at pH 1, and a strong base at pH 14. Following immersion in these solutions for a specified duration, the oil rejection rates of the SHMs were evaluated to confirm their durability and corrosion resistance. As seen in Fig. 6a, throughout the 30-day immersion in artificial brine, the oil rejection of the SHM (mesh size 500) consistently remained over 98.5% and the WCA value below 62°. Plus, even after 30 days of immersion in artificial saltwater, the SHM’s maintained a water flux of up to 51,000 L m^–2^ h^–1^, demonstrating its stable superhydrophilicity and long-term durability. The photographs of the SHM before and after 15 days in brine showed no visible discoloration, rusting, or delamination. As depicted in Fig. 6f, the mesh sheen and surface uniformity were essentially unchanged, consistent with the stable WCA and oil-rejection trends. In Fig. 6d and e, the P-element maps remained homogeneous and of comparable intensity before and after 15 days, with minor phosphorus-depleted patches or localised enrichment that would signal coating loss or corrosive damage. The macroscopic appearance and elemental mapping together indicated that the hybrid conversion layer retained its chemical integrity and surface coverage during prolonged neutral-brine exposure. The oil rejection of the SHM remained constant after being submerged in aqueous solutions with pH values of 1 or 14 for 15 days, as seen in Fig. 6b, demonstrating the device’s exceptional corrosion resistance against both acid and alkali. In addition, the SOM’s ability to withstand corrosion was also examined. Fig. S15 revealed that the SOM consistently maintained an oil rejection rate exceeding 98.1% after a 15-day immersion in an alkaline aqueous solution with a pH of 14. However, in an acidic aqueous solution with a pH of 1, this stability lasted for less than 24 h. Fig. 6c, g, h exhibited the SEM micrographs of the SHM surface, comparing its condition before and after exposure to environments with extreme pH levels. Obviously, the surface of the SHM appeared smooth prior to encountering the corrosive medium, as depicted in Fig. 6c. Upon immersion of the SHM in the acidic corrosive solution with a pH of 1, the surface exhibited signs of corrosion, notably the emergence of etched grain boundaries, as shown in Fig. 6g. This indicated that the surface of the SHM underwent corrosion when exposed to a strong acid. However, compared to the pristine surface, the SHM largely maintained structural integrity, with only a few scattered areas showing signs of corrosion changes. The SHM sample immersed in a strong alkaline solution also exhibited similar phenomena (Fig. 6h). Under the same protocol applied to SHM, Fig. S18 showed that the initially leaf-like architecture of SOM hydrolysed rapidly, evolving into a looser, petal-like morphology after only 7 days of brine immersion. This structural degradation was accompanied by a drop in toluene rejection from 98 to 56% and an increase in water contact angle to 47°, indicating reduced hydrophilicity and weakened separation performance. These changes indicated the limited robustness of SOM relative to SHM. Through the conversion reaction, the dense film that adhered to the surface of the SSM enhanced its anti-corrosion effectiveness. Clearly, the SHM demonstrated outstanding separation efficiency in pH-neutral, acidic, or alkaline conditions, a performance likely attributable to the protective nanohybrid layer of dense conversion film created by the tannic and phosphoric acid. Protected by the conversion film, the stainless steel was capable of preventing direct contact with corrosive substances. Furthermore, relative to materials documented in existing literature, the fabricated SHM exhibited superior attributes in terms of prolonged durability and resistance to corrosion (refer to Fig. S16 and Table S3 [36, 39–47]), indicating its significant promise for real-world application.

### Resistance to abrasion

**Fig. 7:**
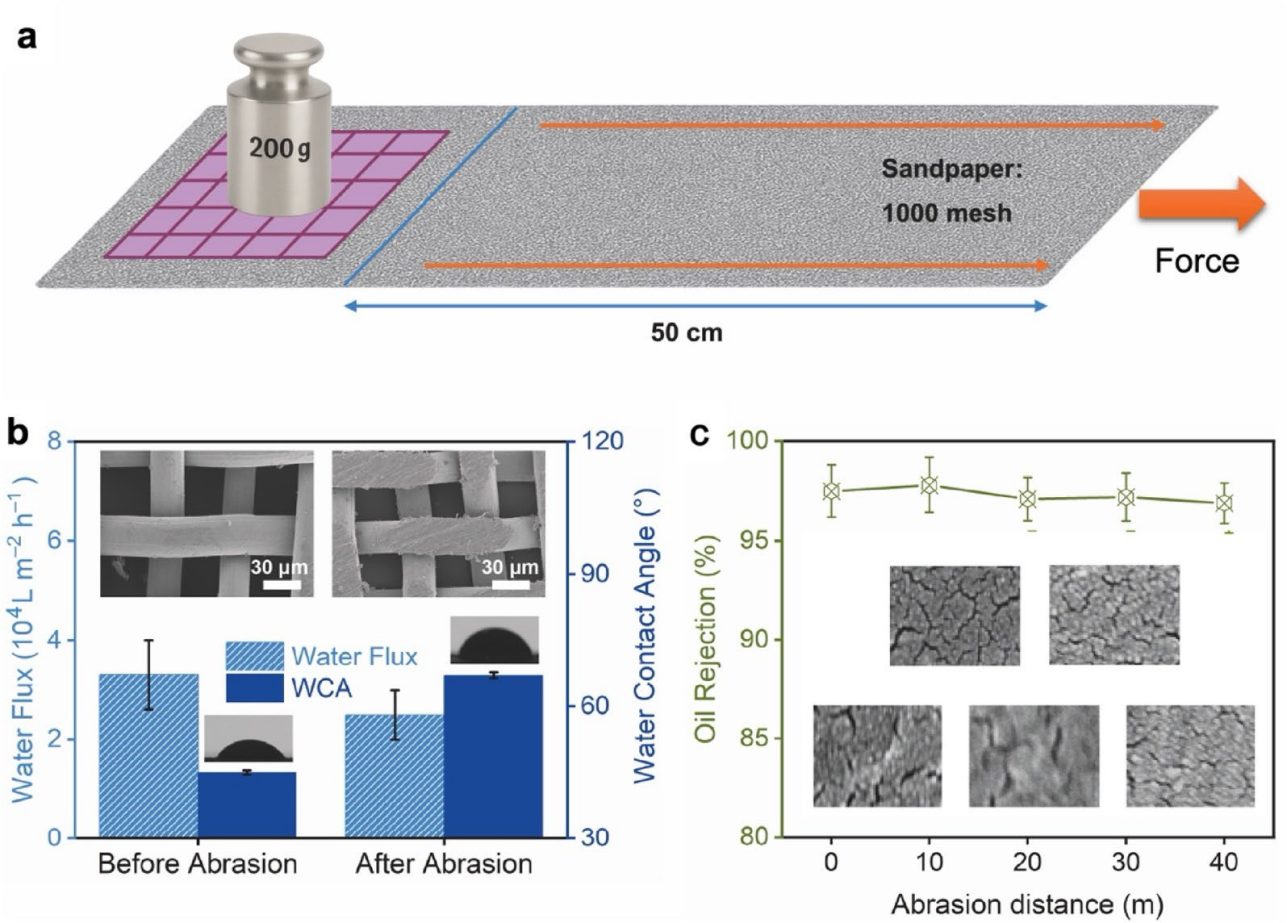
**Abrasion tolerance of the superhydrophilic mesh (SHM).** **a**, Schematic of the reciprocating abrasion test: the mesh was dragged across 1000-grit sandpaper under a 200 g dead load; one stroke covered 50 cm, and the total abrasion distance was the accumulated travel. **b**, Water flux (hatched bars, left axis) and static water contact angle (bars, right axis) before and after abrasion over 20 m under a 200 g load. Insets show SEM overviews of the wire surface pre- and post-abrasion (scale bars, 30 μm). **c**, Oil rejection for a model oil-water mixture versus abrasion distance (0–40 m, 200 g). Rejection remained near-quantitative with minimal drift; insets were representative SEM micrographs of the SHM surface after the indicated distances (scale bars, 500 nm).

A standardised abrasion protocol was implemented to quantify mechanical robustness (Fig. 7a). The SHM was mounted beneath a 200 g dead load and reciprocated back and forth along 1000-grit sandpaper over a 50 cm stroke; the reported abrasion distance corresponded to the cumulative travel. After selected distances, the specimen was retrieved and characterised for surface morphology, static water contact angle (WCA), water flux and oil rejection. As summarised in Fig. 7b, post-abrasion SEM of the wire exterior revealed grooves and scratches, and the WCA increased from ≈ 45° (pristine SHM) to ≈ 67° after 20 m, indicative of partial damage to the outermost hydrophilic layer. Notwithstanding this change in apparent wettability, the permeate water flux changed only modestly, and, critically, the oil rejection remained high. Progressive testing to 40 m (Fig. 7c) confirmed that the SHM maintained > 96.9% oil rejection, demonstrating that the separation function was largely preserved under repeated mechanical loading. Abrasion resistance tests were also performed on the control SOM. As expected, SOM displayed very poor mechanical stability. After only 20 m of abrasion, extensive regions of the mesh surface became severely worn and flattened, accompanied by an increase in water contact angle to 81° and a decrease in toluene rejection to 27%. These findings clearly demonstrated that the oxidised-only SOM layer was highly susceptible to brine exposure and mechanical wear, further underscoring the superior robustness and functional stability provided by the nanohybrid SHM protective layer.

To clarify the nature of the protective layer, cross-sectional analysis was performed. As shown in Fig. S19a–f, SSM, SOM, and SHM all exhibited a similar wire diameter of ≈ 30 μm, and no obvious multilayer contrast was resolved in SEM, most likely because the coating was nanogranular with limited Z-contrast relative to steel. An EDS line scan of phosphorus, originating from phosphate species involved in the conversion reaction, was therefore acquired across the mesh strut. The profile in Fig. S19g showed two narrow regions of high P signal at both edges of the cross section, each extending approximately 1.4–1.5 μm from the surface. These data indicate that the phosphate-tannate/Fe nanohybrid layer formed a conformal shell of about 1.5 μm on a ≈ 30 μm strand, which supports describing the conversion coating as a thin layer. The retained performance can be rationalised by the architecture of the twill-woven substrate (Fig. S17). Abrasion predominantly affects the exterior plane of the wires that first contacted the sandpaper, while the interior aperture walls, which governed imbibition, capillarity and oil repellency during filtration, remained shielded by the weave and therefore retained their superhydrophilic, hybrid coating. Consequently, through-pore wettability and interfacial selectivity were maintained even when the outermost surface exhibited visible scratching and a higher sessile WCA. This decoupling between exterior-face WCA and through-pore separation behaviour explained the minimal drift in rejection with distance. The reciprocating-load test, the before/after SEM micrographs, and the stable flux/rejection metrics established that the SHM possessed strong abrasion tolerance, comparing favourably with the majority of reported systems (Fig. S16), and was well suited for practical deployment where handling and contact wear are unavoidable.

### Self-cleaning and anti-fouling properties

**Fig. 8:**
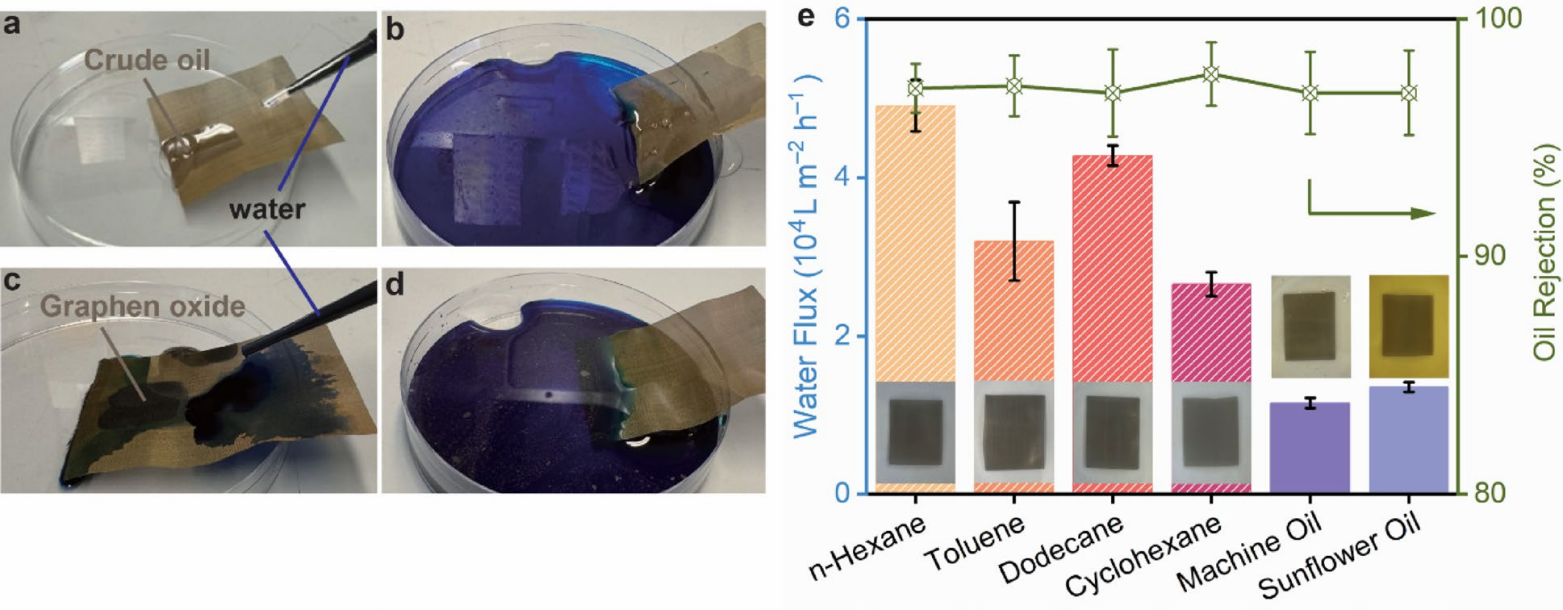
**Anti-fouling/self-cleaning and oil tolerance of the superhydrophilic mesh (SHM).** **a**–**d**, Photographs illustrating the self-cleaning behaviour: when fouled by oil or GO suspensions, the SHM rapidly re-wetted in water and rejected the oil phase, restoring a clean, hydrophilic surface. **e**, Water permeates flux and separation efficiency variations of the SHM after immersing in different oils for 1 h. The inset images showed the SHM immersed in each corresponding oil after 1 h.

Fouling was a primary obstacle to reliable oil-water separation; despite the intrinsic underwater oil repellency of superhydrophilic surfaces, particulate deposition and viscous oil residues can accumulate and degrade performance over time [48, 49]. To probe anti-fouling and self-cleaning, graphene oxide (GO) and crude oil were selected as model microparticulate and oily contaminants. As shown in Fig. 8a–d, a contaminated SHM rapidly re-wetted upon immersion in water, and the adhered foulants were removed by a simple rinse, restoring a clean, hydrophilic surface. This behaviour was consistent with a strongly hydrated interfacial layer on the SHM that suppresses oil adhesion and facilitates contaminant release. Oil tolerance was further quantified by pre-immersing SHMs for 1 h in a panel of oils—*n*-hexane, toluene, dodecane, cyclohexane, machine oil, and sunflower oil—followed by measuring water flux and separation efficiency. As summarised in Fig. 8e, flux values after exposure remained within the same order of magnitude as the pristine state (higher for light hydrocarbons and lower for viscous oils), while the oil rejection stayed high (~ 96.9–97.7%) across all conditions. Relative to its pristine (pre-immersion) state, SHM showed only a ≈ 5,000 L m^–2^ h^–1^ decrease in permeance (≈ 8% flux oss) while maintaining high separation performance, with merely a 0.5% decline in separation efficiency. Insets provided photographs of the SHM after each oil immersion, confirming the absence of persistent wetting or macroscopic residue formation. The rinsing experiments (Fig. 8a–d) and the post-immersion performance (Fig. 8e) both demonstrated that the SHM combined self-cleaning behaviour with chemical/oil tolerance, sustaining both throughput and selectivity after contact with diverse contaminants, which is an essential prerequisite for practical treatment of oily wastewaters.

## Conclusions

This work reported a simple, environmentally benign route to a durable superhydrophilic mesh with strong potential for practical oil-water separation. Using commercial twill-woven stainless-steel mesh as the scaffold, the superhydrophilic hybrid mesh (SHM) was prepared *via* sequential oxidation and tannic-/phosphoric-acid conversion, which endowed the wires with robust hydrophilicity. The resulting meshes delivered high single-pass performance, water fluxes up to 80,000 L m^–2^ h^–1^ and oil rejection > 99%, while maintaining nearly constant selectivity after 30 days in artificial brine and 15 days in strongly acidic or alkaline media, evidencing excellent chemical durability and corrosion resistance. Mechanical robustness was likewise demonstrated by sustained function after 40 m of reciprocating abrasion under a 200 g load. In addition, the SHM rapidly self-cleaned after fouling by graphene oxide or crude oil and, even after 1 h pre-immersion in diverse oils, retained high water selectivity (> 97% oil rejection) with only ≈ 8% permeance loss, consistent with a hydrated, anti-fouling interface. These attributes collectively support the suitability of the modified mesh as a compelling, scalable platform for practical oil-water separation.

## Supplementary Data

Supplementary information: Supplementary Figs. 1–19, Tables 1–3, and Movies 1–2. The online version contains supplementary material available at *Nano Convergence*. Correspondence and requests for materials should be addressed to Professor Yi Huang.

## Supplementary Information

Below is the link to the electronic supplementary material.

Movie S1: WCA videos of superhydrophilic SHM

Movie S2: Demonstration of the oil-water separation process

Supplementary Information

## Data Availability

The data that support the findings of this study are available from the corresponding author upon request.
