# Supplementary material for: Robust functional nanohybrid surface enables long-term oily wastewater remediation
Source: Nano Converg. 2025 Dec 23;12:63. doi: 10.1186/s40580-025-00527-9 (PMC12728155; doi:10.1186/s40580-025-00527-9)
Supplement: Supplementary file 3 — Supplementary Information [file 40580_2025_527_MOESM3_ESM.pdf]

Supplementary Information

Robust Functional Nanohybrid Surface Enables Long-Term Oily Wastewater Remediation

Zheng Chen[1], Xuanyu Zhou[1], Hongyuan Zhang[1], Yuxiang Xue[2], Nurul A. Mazlan[1], Siyu Chen[1], Xiuming Wei[1], Xianfeng Chen[2] and Yi Huang[1,]*

[1]Institute for Materials & Processes, School of Engineering, The University of Edinburgh, Robert Stevenson Road, Edinburgh, EH9 3FB, UK.

[2]Institute for Bioengineering, School of Engineering, The University of Edinburgh, Faraday Building, Edinburgh, EH9 3DW, UK.

***Corresponding author:**

Professor Yi Huang

Tel.: +44-131-6507793

E-mail: Yi.huang@ed.ac.uk

Supplementary figure, table and movie contents:

Fig. S1. Photograph of home-made gravity-driven oil/water separation equipment.

Fig. S2. Surface morphology of stainless-steel mesh (SSM) before and after pre-cleaning.

Fig. S3. Room-temperature reaction failed to yield a coherent oxidation layer, irrespective of duration.

Fig. S4. Reaction at 50 °C produced a continuous oxidation layer within 6 h.

Fig. S5. Oxygen element maps tracking mesh oxidation under different conditions.

Fig. S6. ATR-FTIR evolution of surface hydroxyls during mesh oxidation.

Fig. S7. XRD evolution of iron oxyhydroxide phases under different oxidation conditions.

Fig. S8. Effect of pH control during the conversion step on surface morphology.

Fig. S9. EDS oxygen maps of SHM prepared without precise pH 2.4 control during the conversion.

Fig. S10. Inadequate conversion at R.T. prevented conformal SHM formation.

Fig. S11. Evolution of the conversion layer at 50 °C: from nucleation to continuity and overgrowth.

Fig. S12. Oxygen element maps of SHMs prepared under different conversion conditions.

Fig. S13. ATR-FTIR signatures of SHMs prepared under different conversion conditions.

Fig. S14. XRD evolution of SHMs prepared under different conversion conditions.

Fig. S15. Oil-rejection stability of the oxidised mesh (SOM) under extreme pH.

Fig. S16. Comparison of recycle lifespan and separation efficiency of reported mesh-based oil-water separation materials.

Fig. S17. Pore architecture of a twill-woven stainless-steel mesh.

Fig. S18. Abrasion and chemical robustness of SOM-400.

Fig. S19. Cross-sectional structure and phosphorus distribution across mesh wires.

Table S1. Details from the high resolution XPS spectra of SSM and modifired superhydrophilic mesh.

Table S2. Composition recipe of artificial brine.

Table S3. Comparative analysis of the SHM's performance against other oil-water separation materials documented in the literature.

Movie S1. WCA videos of superhydrophilic SHM.

Movie S2. Demonstration of the separation of a toluene-water mixture.

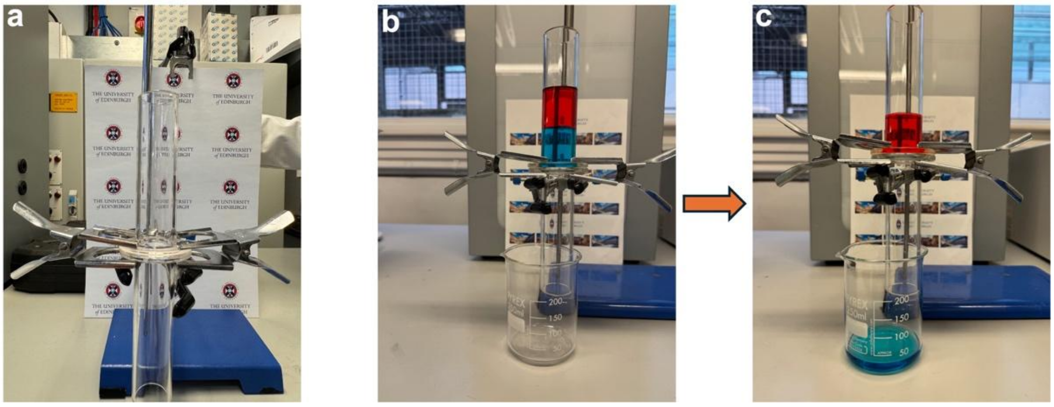

Fig. S1. Oil-water separation equipment and process.

a, Photograph of home-made gravity-driven oil/water separation equipment. **b**–**c**, Loading of a model, density-stratified two-phase mixture (oil red O-dyed oil over methylene blue-dyed water) into the feed column above the SHM.

Additional Analysis: Photograph of the bench-top apparatus (internal diameter 25 mm): the test mesh was clamped at mid-height inside a transparent vertical tube, and separation proceeded under a hydrostatic head defined by the liquid level—no external pump was used.

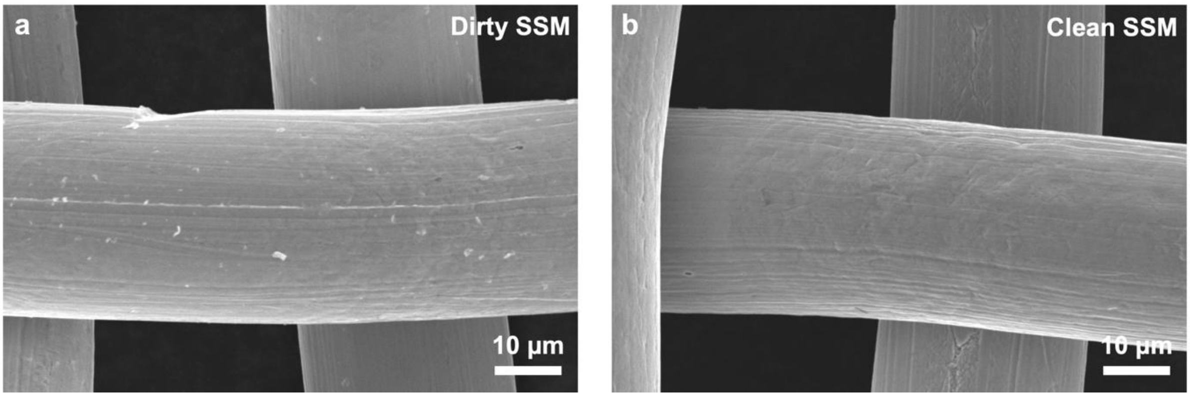

Fig. S2. Surface morphology of stainless-steel mesh (SSM) before and after pre-cleaning.

a, Dirty SSM: scattered particulates and adsorbed residues were visible along the wire surface; **b**, Clean SSM: after the cleaning procedure, the surface appeared smoother with markedly fewer contaminants. Scale bars, 10 µm.

Additional Analysis: Pre-cleaning removed adventitious deposits and revealed the intrinsic metal texture, reducing variability in roughness. This treatment provided a consistent starting interface for subsequent oxidation/conversion, improving coating uniformity and reproducibility.

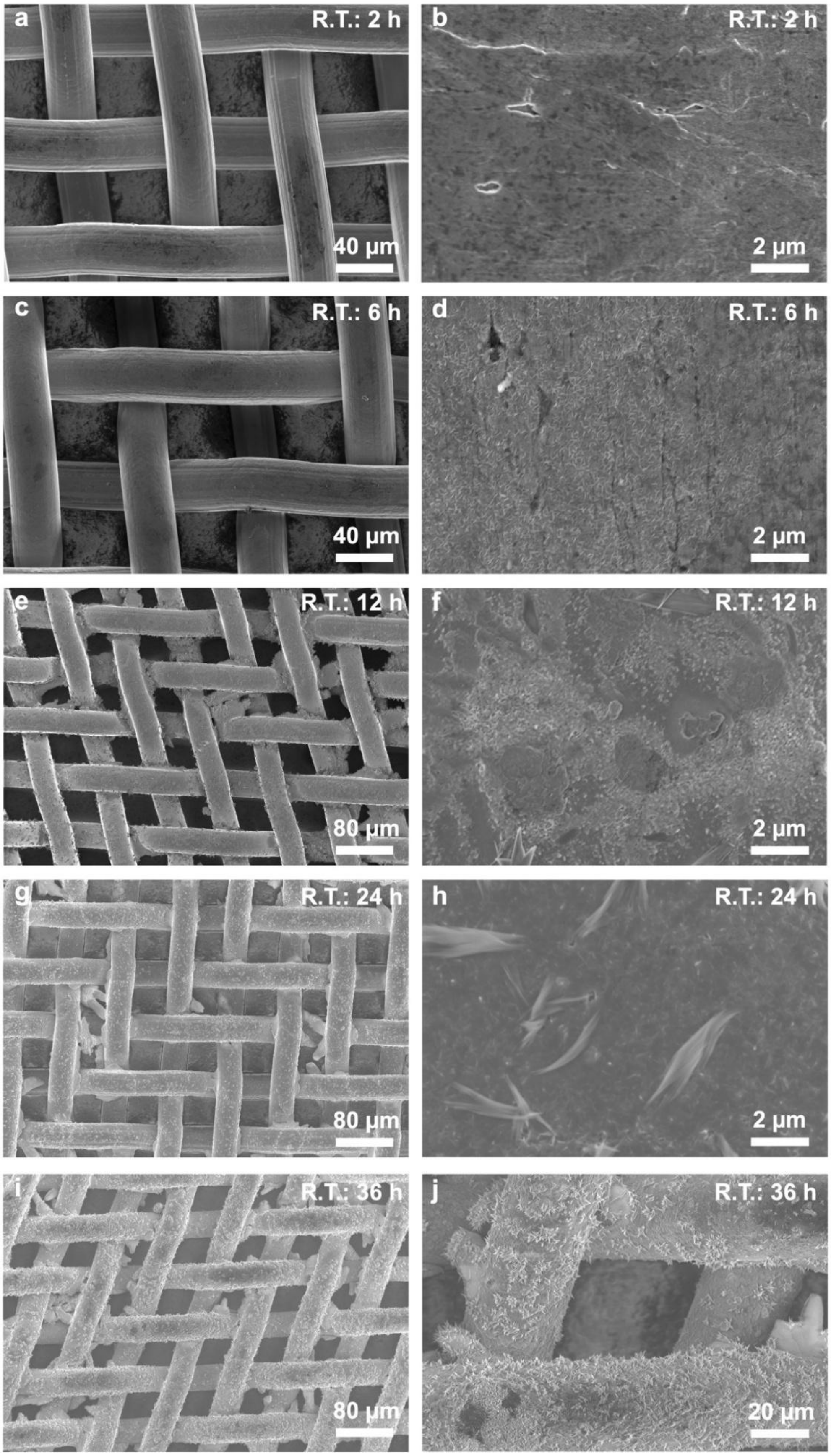
a
R.T.: 2 h
40 μm
b
R.T.: 2 h
2 μm
c
R.T.: 6 h
40 μm
d
R.T.: 6 h
2 μm
e
R.T.: 12 h
80 μm
f
R.T.: 12 h
2 μm
g
R.T.: 24 h
80 μm
h
R.T.: 24 h
2 μm
i
R.T.: 36 h
80 μm
j
R.T.: 36 h
20 μm

Fig. S3. Room-temperature oxidation reaction failed to yield a coherent oxidation layer, irrespective of oxidation duration.

a–j, SEM images of meshes treated at room temperature (R.T.) for 2, 6, 12, 24 and 36 h: left column showed weave-scale views, right column exhibited the corresponding high-magnification morphologies. Short treatments (2–6 h) left the wires largely smooth with sparse etch tracks; extending to 12–24 h produced discontinuous corrosion islands and loose deposits; by 36 h severe pitting and flake-like delamination appeared around the apertures, indicating uncontrolled corrosion rather than a uniform coating. Scale bars as indicated.

Additional Analysis: At R.T., the process oscillated between under-reaction and over-corrosion, never forming a dense, continuous layer even at 36 h. The resulting patchiness and pore-edge damage precluded robust wetting control and stable separation. Consequently, elevated-temperature oxidation was required to drive a rapid and uniform conversion.

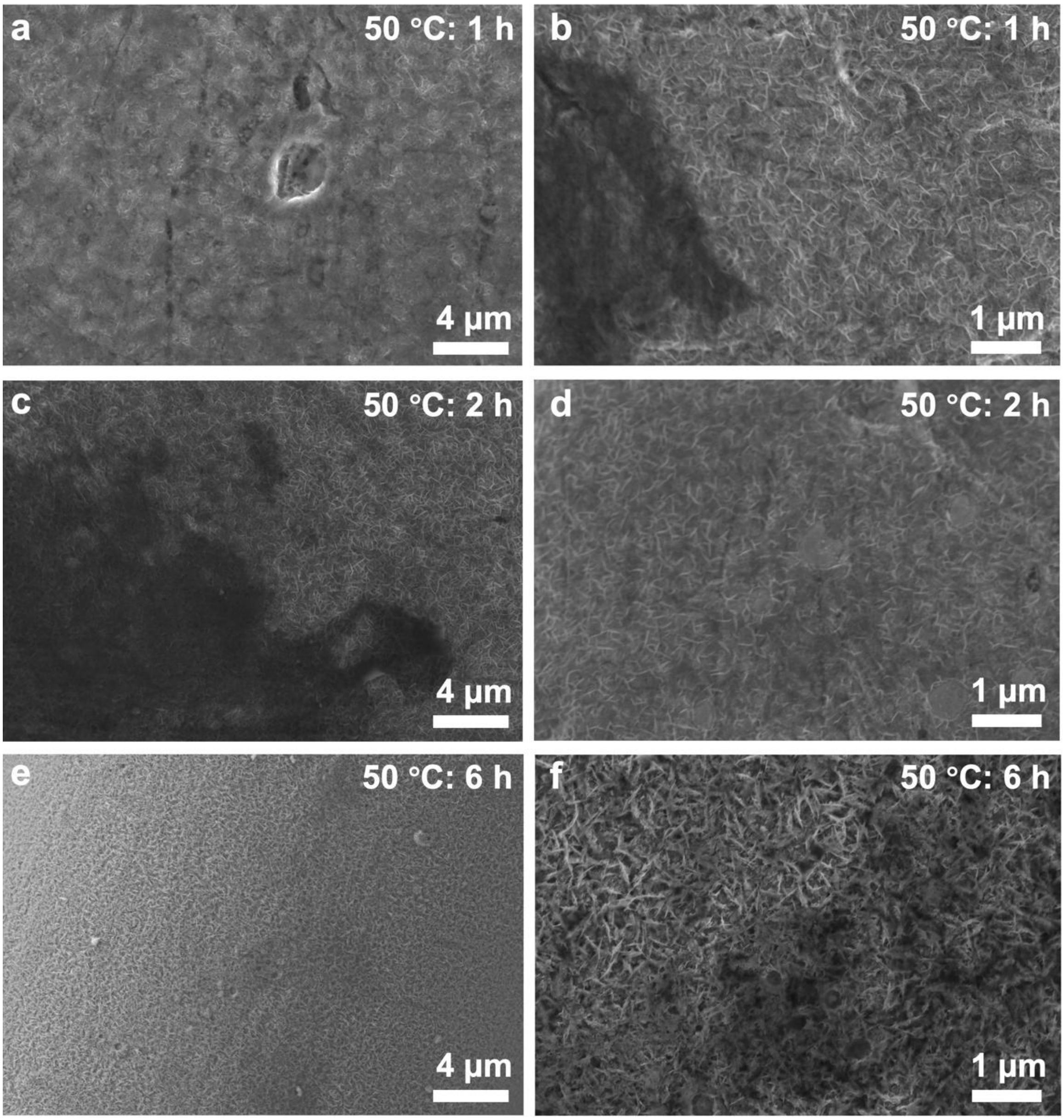

Fig. S4. Oxidation reaction at 50 °C produced a continuous layer within 6 h.

a, **b**, After 1 h, only nascent nanofibrillar features appeared and coverage remained patchy. **c**, **d**, At 2 h, islands coalesced but micron-scale voids persisted. **e**, **f**, By 6 h, the surface was spanned by a dense, interlaced nanofibrillar film indicative of a coherent FeOOH-rich layer. Scale bars: **a**, **c**, **e**, 4 μm; **b**, **d**, **f**, 1 μm.

Additional Analysis: Mild heating accelerated oxidation/coalescence: partial coverage at 1–2 h transitioned to a compact, continuous coating by 6 h. The nanostructure at 6 h was consistent with the wetting and stability improvements observed for the converted meshes.

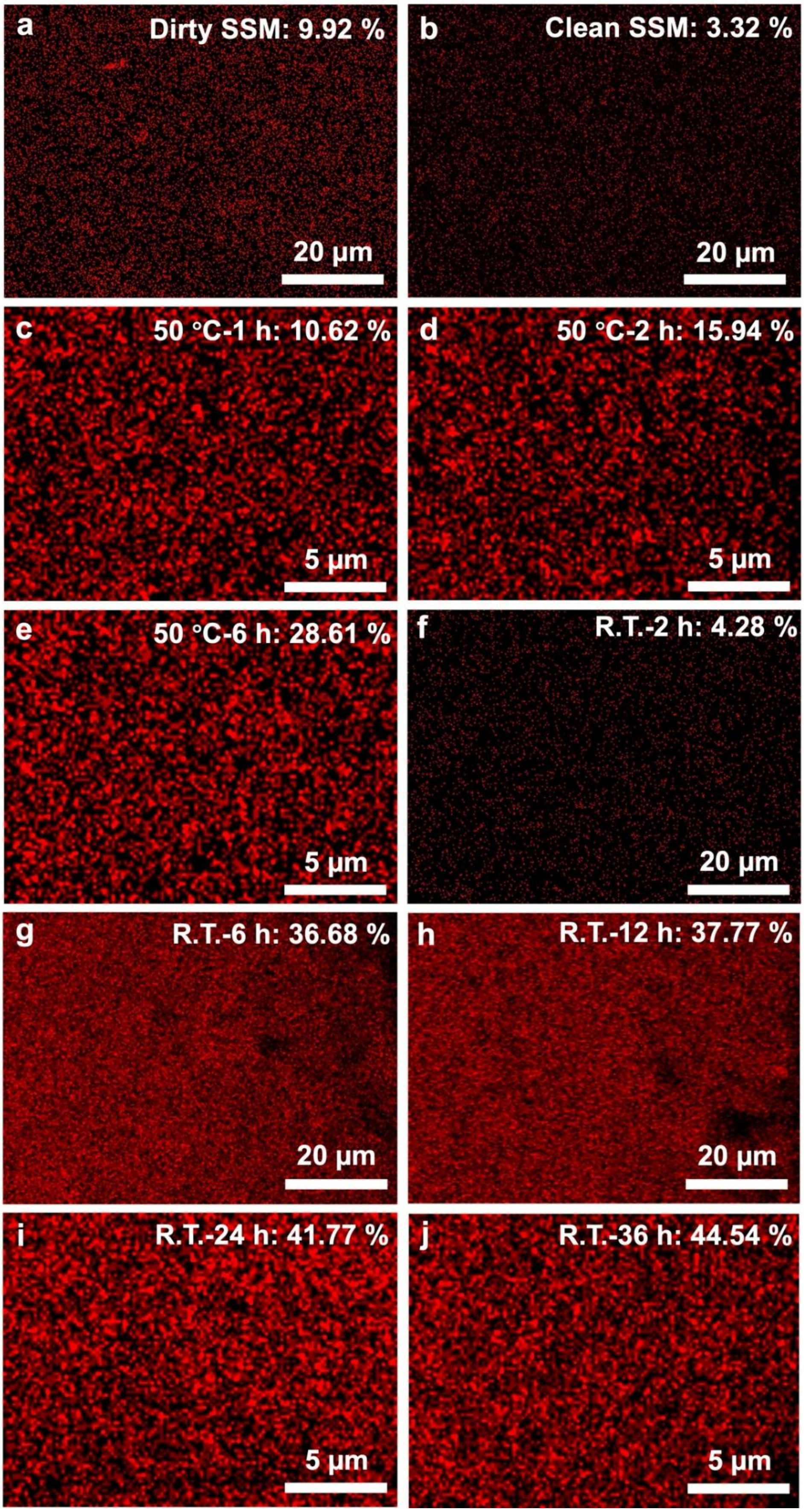
a
Dirty SSM: 9.92 %
20 μm
b
Clean SSM: 3.32 %
20 μm
c
50 °C-1 h: 10.62 %
5 μm
d
50 °C-2 h: 15.94 %
5 μm
e
50 °C-6 h: 28.61 %
5 μm
f
R.T.-2 h: 4.28 %
20 μm
g
R.T.-6 h: 36.68 %
20 μm
h
R.T.-12 h: 37.77 %
20 μm
i
R.T.-24 h: 41.77 %
5 μm
j
R.T.-36 h: 44.54 %
5 μm

Fig. S5. EDS oxygen maps tracking mesh oxidation under different conditions.

a, **b**, Baselines for a dirty SSM (9.92%) and a cleaned SSM (3.32%). **c**–**e**, SOM prepared at 50 °C for 1, 2, and 6 h showed progressively stronger O signal (10.62%, 15.94%, 28.61%). **f**–**j**, SOM prepared at room temperature (R.T.) for 2, 6, 12, 24, and 36 h yielded 4.28%, 36.68%, 37.77%, 41.77%, 44.54% O, respectively. Red pixels denoted the oxygen EDS signal; percentages were areal O fractions extracted from the maps.

Additional Analysis: Relative to pristine SSM, the modified SOM exhibited a markedly denser and more uniform O signal (red), indicating successful formation of an oxygen-rich surface layer and more homogeneous coverage.

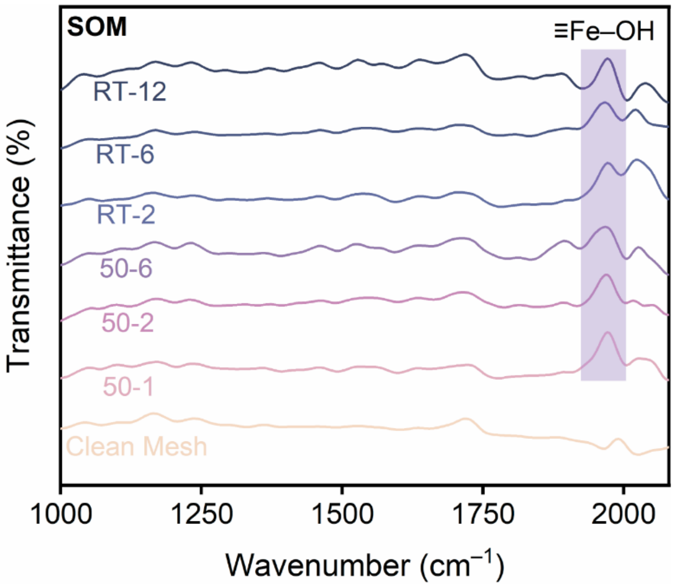

Fig. S6. ATR-FTIR evolution of surface hydroxyls during mesh oxidation.

Spectra of SOM prepared at R.T. (2–12 h) and at 50 °C (1–6 h), compared with the clean mesh baseline. The shaded region highlighted the band at ≈1967 cm^{-1}, assigned to surface ≡Fe–OH groups; its intensity increased with either temperature or duration, indicating progressive hydroxylation.

Additional Analysis: The ≈1967 cm^{-1} Fe–OH feature was negligible on the clean mesh, faint after short R.T. treatments, and most pronounced for 50 °C-6 h, evidencing accelerated formation of an FeOOH-rich layer at elevated temperature. This trend supported 50 °C-6 h as a minimum condition for a continuous conversion coating.

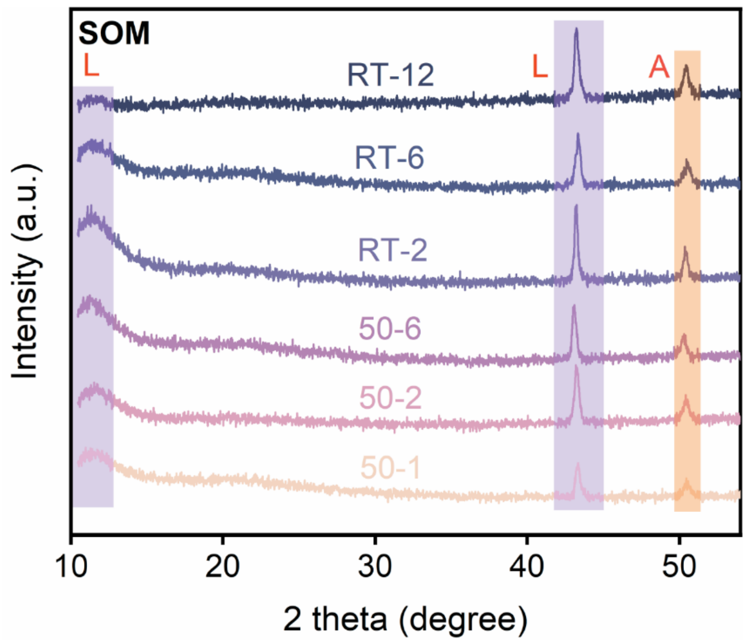

Fig. S7. XRD evolution of iron oxyhydroxide phases under different oxidation conditions. XRD patterns for SOM prepared at R.T. (RT-2 h, RT-6 h, RT-12 h) and at 50 °C (50-1 h, 50-2 h, 50-6 h). Shaded bands marked reflections characteristic of lepidocrocite (γ-FeOOH, L) and akaganeite (β-FeOOH, A); the L features at low angle and ~40° and the A feature near ~50° were highlighted.

Additional Analysis: Peak sharpening and intensity growth with increasing temperature/time—most evident for 50 °C-6 h—indicated improved crystallinity and a higher fraction of FeOOH phases. R.T. treatments yielded broader, weaker L/A peaks, consistent with incomplete phase development.

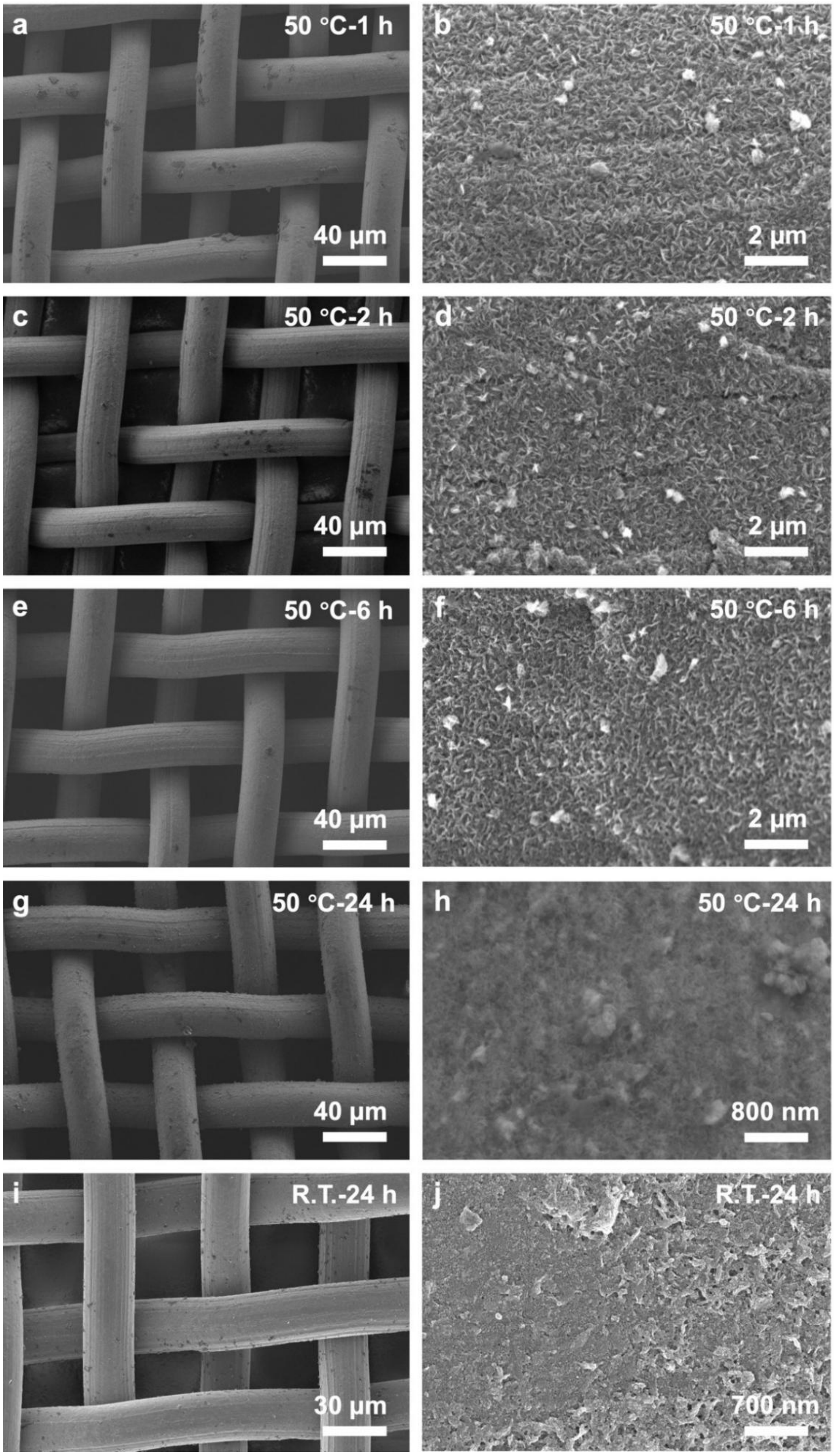
a
50 °C-1 h
40 μm
b
50 °C-1 h
2 μm
c
50 °C-2 h
40 μm
d
50 °C-2 h
2 μm
e
50 °C-6 h
40 μm
f
50 °C-6 h
2 μm
g
50 °C-24 h
40 μm
h
50 °C-24 h
800 nm
i
R.T.-24 h
30 μm
j
R.T.-24 h
700 nm

Fig. S8. Effect of pH control during the conversion step on surface morphology.

SEM images of meshes processed in the "conversion" step without precise pH 2.4 adjustment: 50 °C for (**a**, **b**) 1 h, (**c**, **d**) 2 h, (**e**, **f**) 6 h, (**g**, **h**) 24 h and R.T. for (**i**, **j**) 24 h. The weave-scale views (left column) and high-magnification surfaces (right column) showed that the coatings remained SOM-like across conditions, lacking the continuous nanogranular film characteristic of properly converted SHM. Scale bars: **a**, **c**, **e**, **g**, 40 µm; **i**, 30 µm; **b**, **d**, **f**, 2 µm; **h**, 800 nm; **j**, 700 nm.

Additional Analysis: In the absence of tight pH 2.4 control, phosphate/tannate complexation with surface Fe^{3+} was inefficient, leaving a fibrillar FeOOH texture akin to SOM rather than a conformal SHM layer. Even prolonged treatment (50 °C-24 h or R.T.-24 h) did not yield a dense, continuous coating, underscoring the necessity of precise pH regulation for successful conversion.

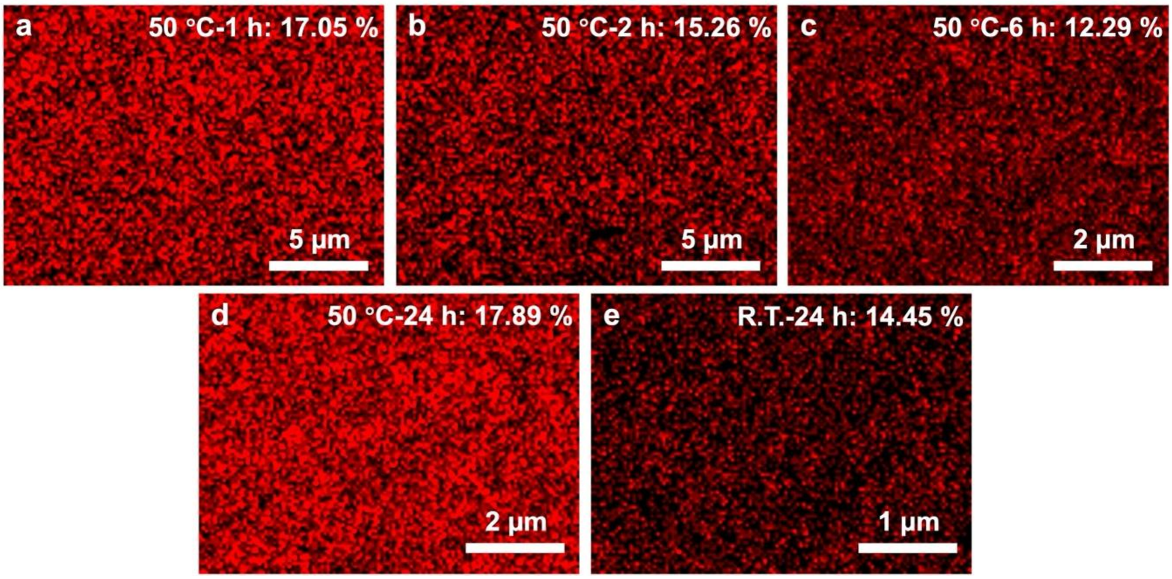

Fig. S9. EDS oxygen maps of SHM prepared without precise pH control during the conversion step.

a–**e**, Oxygen element maps for meshes converted at 50 °C for 1 h (17.05%), 2 h (15.26%), 6 h (12.29%), 24 h (17.89%), and at R.T. for 24 h (14.45%). Red pixels denoted the O signal; percentages were the areal O fractions extracted from the maps. Scale bars: 5 μm in **a**, **b**; 2 μm in **c**, **d**; 1 μm in **e**.

Additional Analysis: In the absence of tight pH regulation, oxygen enrichment remained modest and non-monotonic (≈12–18%) with pronounced spatial heterogeneity, unlike the uniformly O-deficient coatings obtained at pH = 2.4. This behaviour indicated inefficient phosphate-tannate complexation and a surface that remained largely FeOOH-like, consistent with the SOM-like morphology observed by SEM.

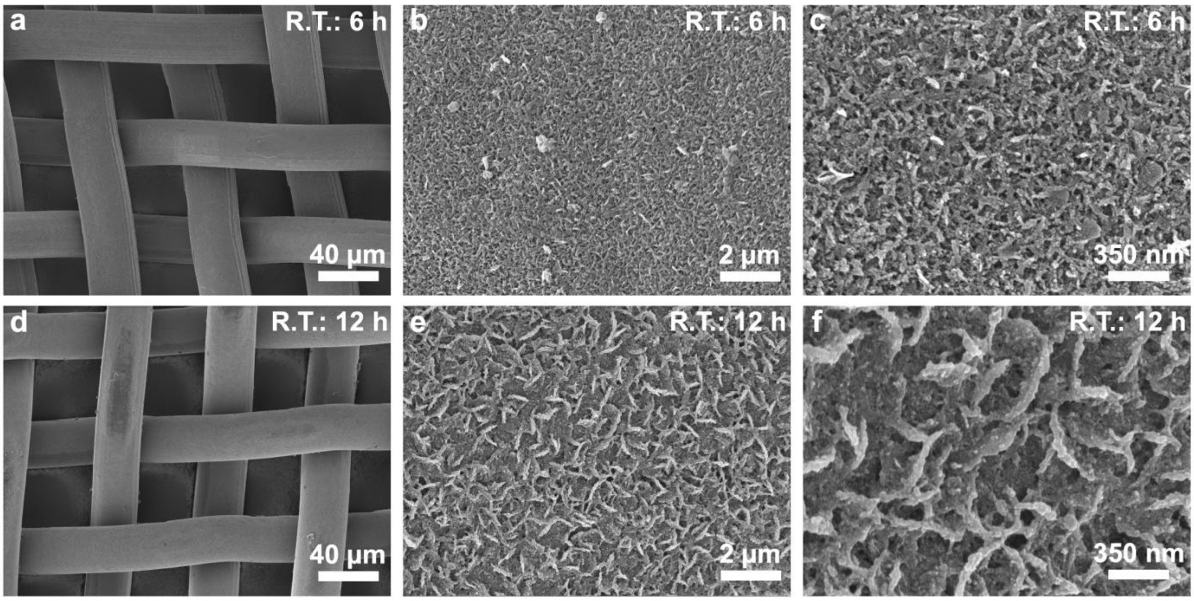

Fig. S10. Inadequate conversion at R.T. prevented conformal SHM formation.

a–**c**, Mesh treated at R.T.-6 h: weave-scale view (**a**) and higher-magnification surfaces (**b**, **c**) showed a sparse, worm-like FeOOH texture with abundant sub-micrometre voids. **d**–**f**, Even after R.T.-12 h, the surface evolved into a more corrugated fibrillar network (**e**, **f**) but remained highly porous and discontinuous. Scale bars: **a**, **d**, 40 µm; **b**, **e**, 2 µm; **c**, **f**, 350 nm.

Additional Analysis: R.T. processing—whether 6 h or 12 h—failed to form the dense, nanogranular film characteristic of functional SHM. The persistent porosity and fissures implied poor wetting stability and mechanical integrity, underscoring the need for elevated temperature/controlled pH for successful conversion.

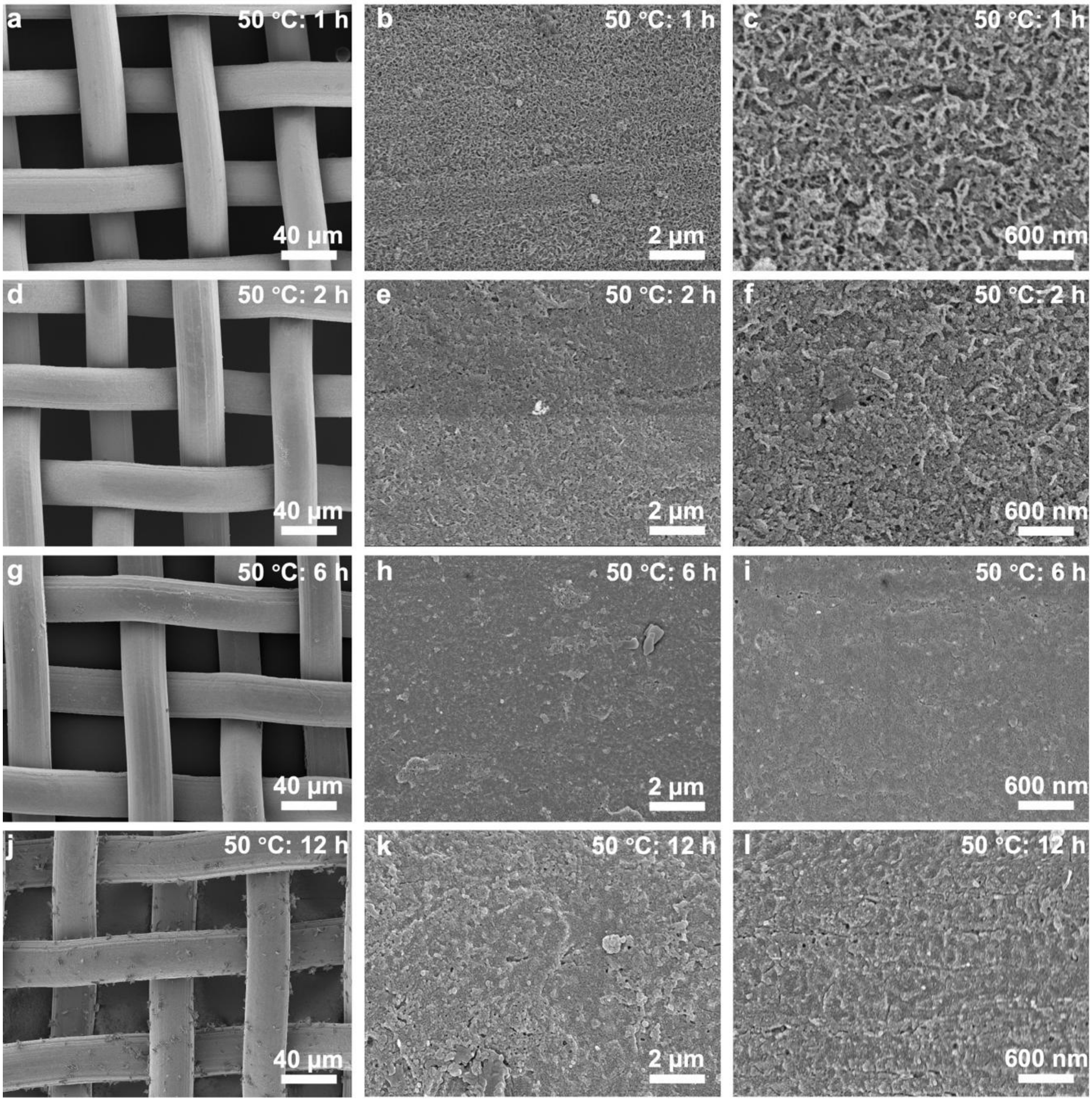

Fig. S11. Evolution of the conversion layer at 50 °C: from nucleation to continuity and overgrowth. SEM images of meshes converted at 50 °C for (**a**–**c**) 1 h, (**d**–**f**) 2 h, (**g**–**i**) 6 h and (**j**–**l**) 12 h. At 1–2 h the surface showed sparse nanofibrillar islands and incomplete coverage; by 6 h a dense, continuous nanogranular film spanned the wires; at 12 h the coating coarsened into thicker aggregates with local pore infilling and crust-like features. Scale bars: left column 40 μm, middle 2 μm, right 600 nm.

Additional Analysis: Mild heating to 50 °C accelerated conversion, yielding an optimal, conformal layer by 6 h. Prolonging to 12 h drived over-reaction and morphological coarsening, which was detrimental to coating uniformity and transport.

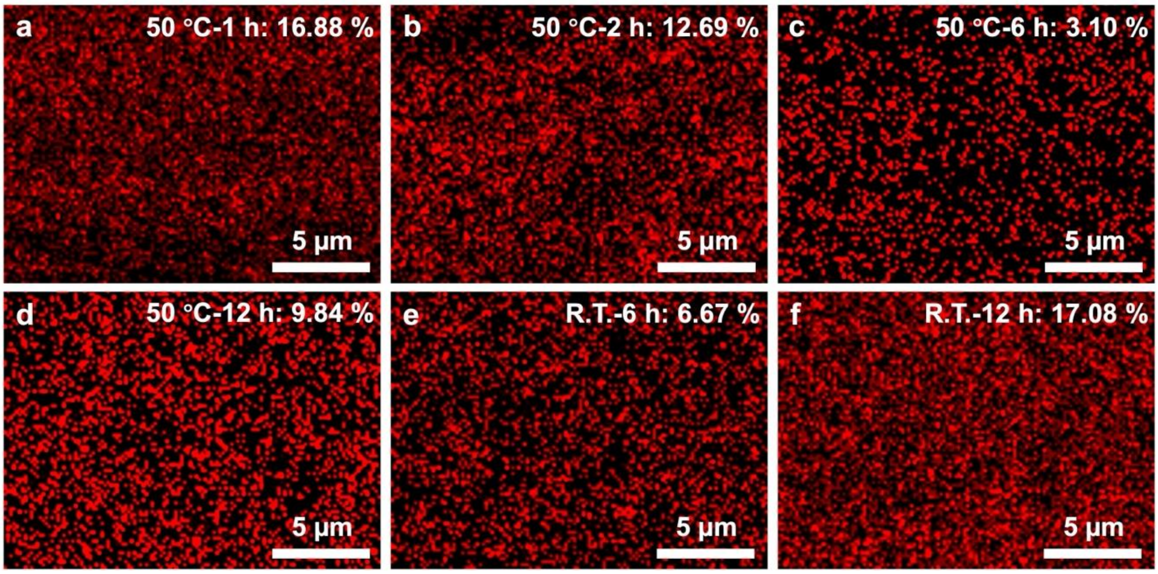

Fig. S12. EDS oxygen element maps of SHMs prepared under different conversion conditions. **a**–**d**, SHM prepared at 50 °C for 1 h (16.88%), 2 h (12.69%), 6 h (3.10%), 12 h (9.84%); **e**,**f**, SHM prepared at R.T. for 6 h (6.67%) and 12 h (17.08%). Red pixels denoted the O signal; percentages were the areal O fractions extracted from each map. Scale bars, 5 μm.

Additional Analysis: The O signal showed a monotonic dependence on time and temperature—dropping to a minimum at 50 °C-6 h and rising again for prolonged or R.T. treatments. This indicated that EDS O intensity alone was a proxy for coating quality: the optimised 50 °C-6 h condition, lower in apparent O fraction, corresponded to a denser, more continuous phosphate-tannate/Fe layer (attenuating the O signal).

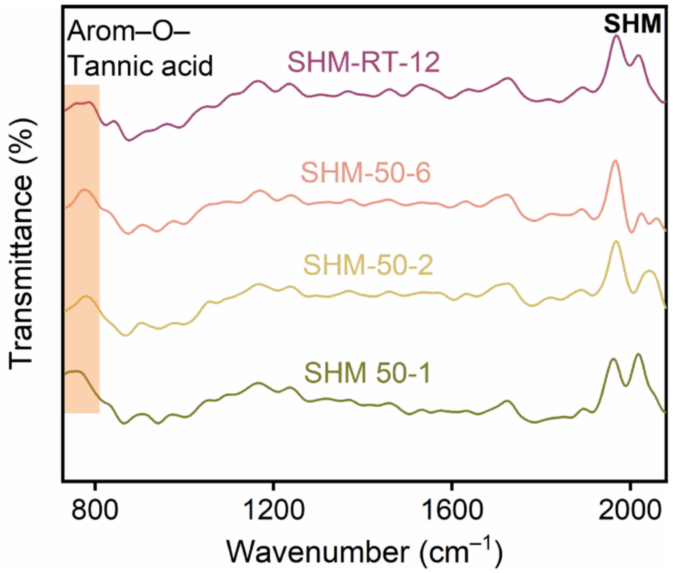

Fig. S13. ATR-FTIR signatures of SHMs prepared under different conversion conditions.

Spectra of meshes converted at 50 °C for 1, 2, and 6 h and at R.T. for 12 h. The shaded band at ≈780–810 cm^{-1} corresponded to the aromatic O^{-} vibration of tannic acid, evidencing tannic acid (TA) incorporation into the hybrid coating; the high-wavenumber feature near ~1965–2000 cm^{-1} reflects surface ≡Fe–OH groups.

Additional Analysis: The TA-associated band was most pronounced for 50 °C-6 h, whereas the RT-12 h sample showed a weaker, less defined signature, indicating inefficient complexation at ambient conditions. Together with the persistent ≡Fe–OH feature, these trends supported that elevated temperature and sufficient time (≈6 h) were required to form a robust phosphate-tannate/Fe network.

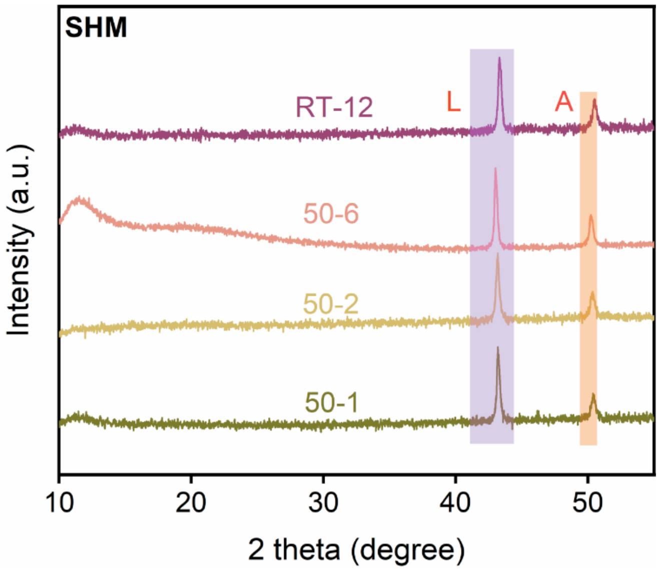

Fig. S14. XRD evolution of SHMs prepared under different conversion conditions.

XRD patterns for meshes converted at 50 °C for 1, 2, and 6 h and at R.T. for 12 h. Shaded bands marked reflections characteristic of lepidocrocite (γ-FeOOH, L) and akaganeite (β-FeOOH, A) (Cu Kα).

Additional Analysis: Relative to the SOM reference (see Fig. S7), the SHM patterns lacked the low-angle lepidocrocite peak (~10°), and the reflections at ~43° (L) and ~51° (A) were uniformly attenuated across conditions. This suppression indicated a reduced abundance and/or crystallinity of surface FeOOH after conversion, consistent with coverage by the phosphate-tannate hybrid layer.

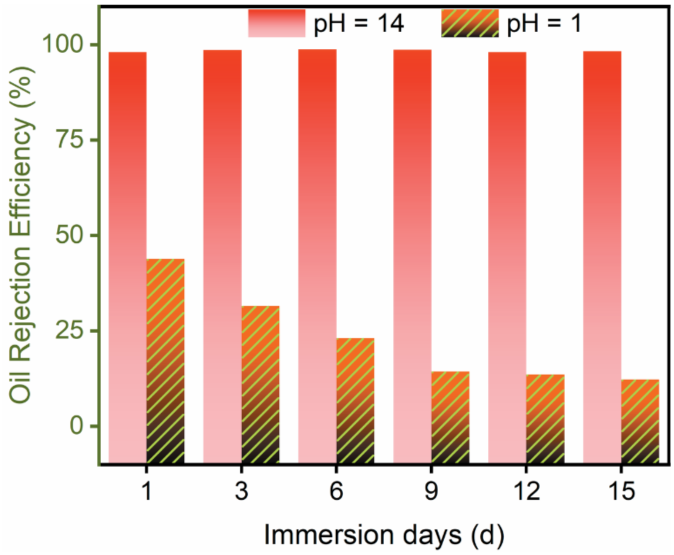

Fig. S15. Oil-rejection stability of the oxidised mesh (SOM) under extreme pH.

Alteration in the oil rejection percentage (%) of the SOM while submerged in aqueous solutions with pH values of 1 and 14 over a period of 15 days.

Additional Analysis: Across 15 days of immersion in pH of 1 (hatched) and pH of 14 (gradient) solutions, the SOM retained near-quantitative oil rejection (≈98.1–98.8%) with no systematic decay, evidencing excellent chemical robustness under extreme pH conditions.

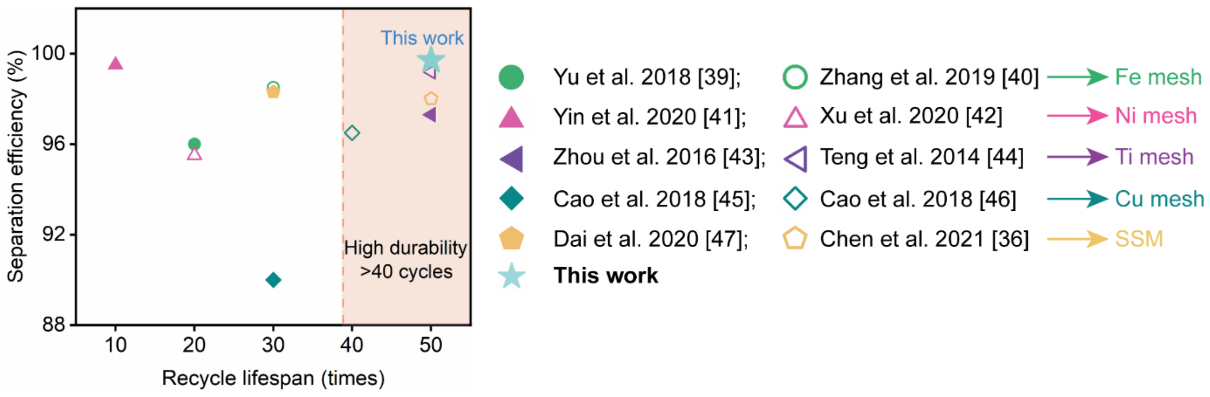

Fig. S16. Comparison of recycle lifespan and separation efficiency of reported mesh-based oil-water separation materials. SSM denotes Stainless Steel mesh.

Scatter plot of reported systems showing separation efficiency versus number of reuse cycles. The shaded region marked the high-durability regime (>40 cycles). Star markers indicated the SHM from this work. All references have been cited in the main text.

Additional Analysis: Relative to most literature examples that operated for ~10–35 cycles, the SHM resided in the >40-cycle region while maintaining ~97–99% separation efficiency. The rightward position at comparable or higher efficiency indicated enhanced durability without sacrificing selectivity, underscoring good prospects for practical use.

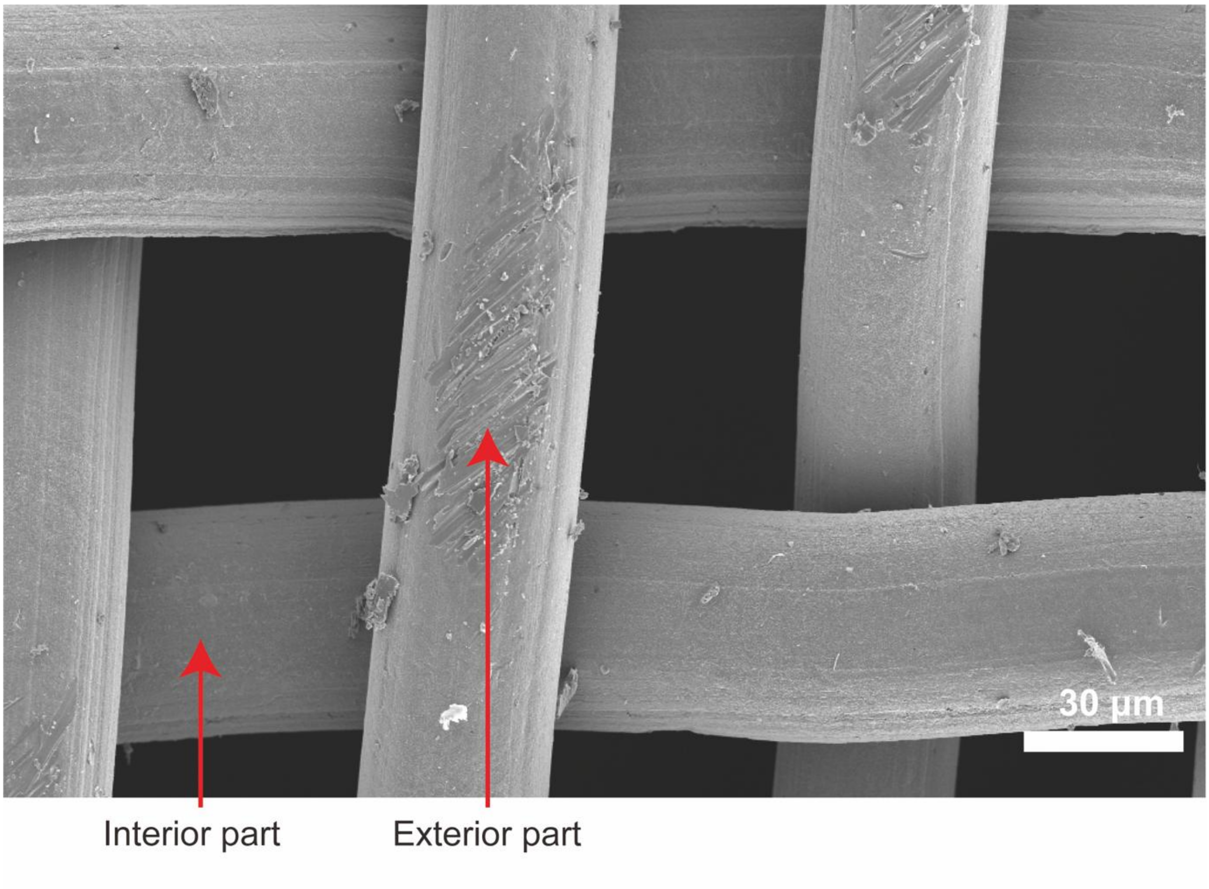

Fig. S17. Pore architecture of a twill-woven stainless-steel mesh.

SEM micrograph highlighting the exterior wire (feed-facing plane) and the interior wire that lines the aperture wall (red arrows). Scale bar, 30 µm.

Additional Analysis: The distinction between exterior and interior surfaces clarified the origin of wetting and transport asymmetry: coatings applied from the feed side primarily modified the exterior plane, whereas permeation and fouling were governed by the interior-lined channels. Effective functionalization must therefore penetrate into the aperture to ensure uniform wettability and stable separation.

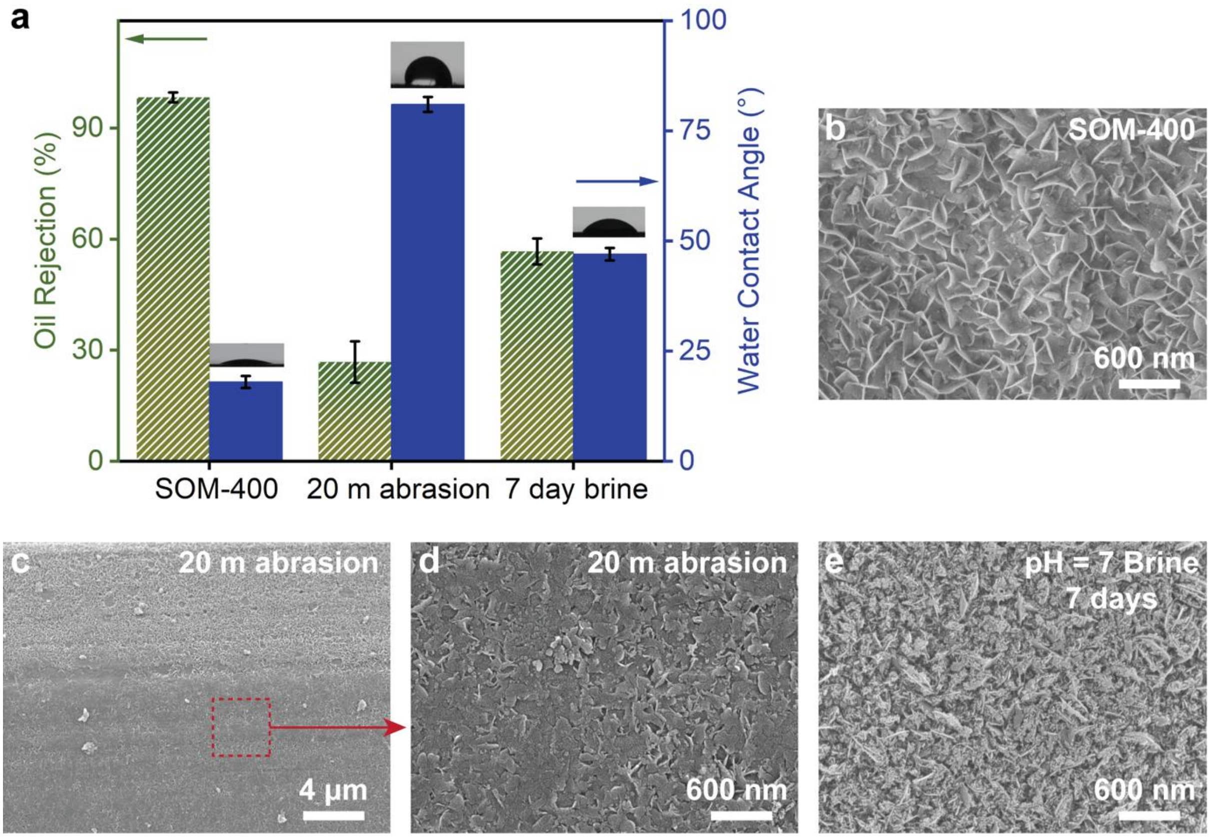

Fig. S18. Abrasion and chemical robustness of SOM-400.

a, Oil rejection (green, left axis) and static WCA (blue, right axis) for SOM-400 as-prepared, after 20 m abrasion (200 g on 1000-grit), and after 7 days in pH 7 brine; insets: representative droplet images. **b**, SEM of the as-prepared SOM-400 surface. **c**, Low-magnification SEM of SOM-400 after 20 m abrasion (boxed area corresponds to high-magnification scanned area). **d**, High-magnification SEM of the abraded region. **e**, SEM of SOM-400 after 7 days immersion in pH 7 brine.

Additional Analysis: Mechanical wear markedly degraded the SOM surface; after 20 m of abrasion, the WCA rose, and oil-rejection dropped, consistent with the SEM evidence of ploughing scars and disrupted wrinkle networks. Subsequent immersion in neutral brine partly restored wettability and separation, but the performance remained below the pristine state, and the surface exhibited corrosion-like granulation, underscoring the limited robustness of SOM relative to SHM.

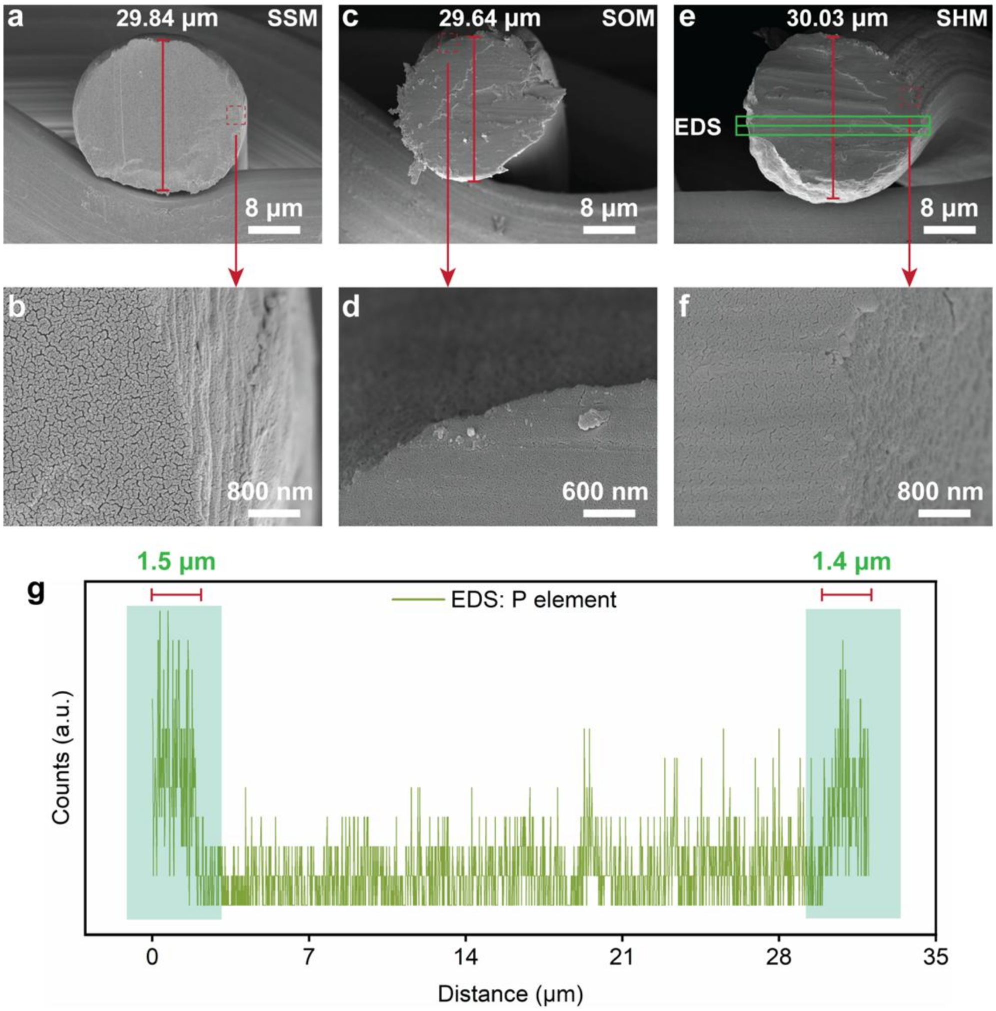

Fig. S19. Cross-sectional structure and phosphorus distribution across mesh wires.

a, **b**, Bare SSM: cross-section (**a**: wire diameter of 29.84 μm) and (**b**) high-magnification rim view. **c**, **d**, After oxidation (SOM): cross-section (**c**: wire diameter of 29.64 μm) and (**d**) rim morphology. **e**, **f**, After conversion (SHM): cross-section (**e**: wire diameter of 30.03 μm) with the green line indicating the EDS line scan path and (**f**) corresponding rim view. **g**, EDS line-scan of the P element across the wire diameter in (**e**), showing P-rich edge bands of ~1.5 μm and ~1.4 μm at the two sides.

Additional Analysis: Cross-sectional SEM indicated minimal change in wire diameter from SSM to SOM to SHM, consistent with a surface-confined modification rather than bulk corrosion. In SHM, the rim was compact compared with the etched SOM, and the EDS line scan showed symmetric phosphorus enrichment bands of about 1.5 μm at both outer surfaces, evidencing a conformal phosphate-tannate conversion layer. The absence of phosphorus in the core confirmed that the treatment formed a protective shell while preserving the metallic interior.

Table S1. Details from the high-resolution XPS spectra of clean mesh and modified superhydrophilic mesh.

Mesh Type	Peak Location (eV)			Atomic Percentage (%)			
	C1s	O1s	P2p	C	O	P	Other
SSM	285.10	531.89	N/A	55.51	38.25	N/A	6.24
SOM	285.08	531.71	N/A	31.06	58.16	N/A	10.78
SHM	285.41	532.02	133.53	57.75	38.80	1.79	1.66

Table S2. Composition recipe of artificial brine (pH 7).

Salts	NaCl	$MgCl_2$	$CaCl_2$	KCl
Concentration (g/L)	0.3	12.0	1.1	0.7

Table S3. Comparative analysis of the SHM's performance against other oil-water separation materials documented in the literature.

Substrate type	Modified materials	Recycle lifespan times	Abrasion resistance	Separation efficiency	Chemical resistance	Ref
Fe mesh	Bi_2O_3/Fe_2O_3	20	20 m under 30 g	96%	N/A	[39]
Fe mesh	$PDA/PEI/NiSO_4$	30	N/A	>98%	pH 4 or 10 up to 2 h	[40]
Ni mesh	NiS nanorods	20	0.5 m under 100 g	>95%	High temp. 200 ℃	[41]
Ni mesh	NiO/Ni-SA	10	N/A	>99%	300 ℃, acid resistance	[42]
Ti mesh	TiO_2 nanowires	50	N/A	98%	pH 4 to 11 up to 2 h	[43]
Ti mesh	PEDOT-PSS hydrogel	50	N/A	99.3%	80 ℃ up to 3 days, acid/base resistance	[44]
Cu mesh	Polyurethane/SiO_2	40	200 times peel off under 100 g	96.5%	Near 100 ℃, pH 4 to 11	[45]
Cu mesh	Candle ashes/ Carbon Nps	30	2 m under 5 N pressure	90%	pH 1 to 12, 375 ℃	[46]
SSM	Graphene oxide/ $CaCO_3$	30	25.4 ± 2.6 GPa	>98%	pH 3 to 11 up to 24 h	[47]
SSM	ZIF-L	50	N/A	>99%	pH 4 to 14 up to 6 h	[36]
**SSM**	**Tannic acid/H_3PO_4**	**50**	**20 m under 200 g**	**>99.7%**	**pH 1 to 14 up to 15 days**	**This work**

All references have been cited in the References section of the main text.

Movie S1. WCA videos of superhydrophilic SHM (see separate Movie S1)

Movie S2. Demonstration of the toluene-water separation process. (see separate Movie S2)
